# Genome-wide association analysis provides insights into the genetic basis of photosynthetic responses to low-temperature stress in spring barley

**DOI:** 10.3389/fpls.2023.1159016

**Published:** 2023-06-06

**Authors:** Ammar Elakhdar, Jan J. Slaski, Takahiko Kubo, Aladdin Hamwieh, Guillermo Hernandez Ramirez, Aaron D. Beattie, Ludovic J.A. Capo-chichi

**Affiliations:** ^1^ Field Crops Research Institute, Agricultural Research Center, Giza, Egypt; ^2^ Institute of Genetic Resources, Faculty of Agriculture, Kyushu University, Fukuoka, Japan; ^3^ Bio Industrial Services Division, InnoTech Alberta Inc., Vegreville, AB, Canada; ^4^ International Center for Agriculture Research in the Dry Areas (ICARDA), Giza, Egypt; ^5^ Department of Renewable Resources, Faculty of Agriculture, Life and Environmental Sciences, University of Alberta, Edmonton, AB, Canada; ^6^ Department of Plant Sciences, College of Agriculture and Bioresources, University of Saskatchewan, Saskatoon, SK, Canada

**Keywords:** *Hordeum vulgare* L., chlorophyll fluorescence, photosystem II photochemistry, quantitative trait nucleotides (QTNs), Mixed linear model (MLM), Abscisic acid (ABA) signaling, protein kinase, post-transcription modification

## Abstract

Low-temperature stress (LTS) is among the major abiotic stresses affecting the geographical distribution and productivity of the most important crops. Understanding the genetic basis of photosynthetic variation under cold stress is necessary for developing more climate-resilient barley cultivars. To that end, we investigated the ability of chlorophyll fluorescence parameters (F_V_F_M,_ and F_V_F_0_) to respond to changes in the maximum quantum yield of Photosystem II photochemistry as an indicator of photosynthetic energy. A panel of 96 barley spring cultivars from different breeding zones of Canada was evaluated for chlorophyll fluorescence-related traits under cold acclimation and freeze shock stresses at different times. Genome-wide association studies (GWAS) were performed using a mixed linear model (MLM). We identified three major and putative genomic regions harboring 52 significant quantitative trait nucleotides (QTNs) on chromosomes 1H, 3H, and 6H for low-temperature tolerance. Functional annotation indicated several QTNs were either within the known or close to genes that play important roles in the photosynthetic metabolites such as abscisic acid (ABA) signaling, hydrolase activity, protein kinase, and transduction of environmental signal transduction at the posttranslational modification levels. These outcomes revealed that barley plants modified their gene expression profile in response to decreasing temperatures resulting in physiological and biochemical modifications. Cold tolerance could influence a long-term adaption of barley in many parts of the world. Since the degree and frequency of LTS vary considerably among production sites. Hence, these results could shed light on potential approaches for improving barley productivity under low-temperature stress.

## 1 Introduction

Environmental factors, particularly those affecting temperature and availability of water are the main factors of in plant growth and development. The low-temperature polar regions and Oceans cover about 80% of the Globe’s land. One-third only of the global lands are free of ice, and 42% of this area is commonly under temperatures below -20°C (Junttila and Robberecht, 1999). In such regions, plants require specific processes to survive exposure to low temperatures. Most plants have developed a degree of cold tolerance, which is usually based on a combination of the length of exposure to cold stress and the minimum temperature experienced (Janska et al., 2010). Therefore, the growth and development of plants are affected by temperature variations in the majority of temperate regions on Earth (Nilsen et al., 1996). The problem is expected to expand due to climatic changes, particularly in Northern Europe and Canada. Cold stress impacts plant growth and crop productivity, causing substantial yield reductions (Junttila and Robberecht, 1999). Plants vary in their responses to freezing (<0°C) and chilling (0-15°C) temperatures (Browse and Xin, 2001). Damage appears when low temperatures overlap with sensitive stages of plant growth.

In barley (*Hordeum vulgare L*.), yield and grain quality are the key challenges in maintaining the constant growth of the agriculture industry. Though significant advances have been made in the genetic gains for yield and grain quality in barley, the crop rarely reaches its full yield potential because of seasonal variations including the low temperatures prevailing early and late in the growing season. Barley is mainly classified into three types of growth habits: spring, winter, and facultative. Winter barley is sown in the autumn because it requires a cold period (vernalization) to flower. Facultative types do not require vernalization, some lines possess levels of low-temperature tolerance comparable to winter barley. While, spring barleys are sown in the spring and do not require vernalization, and possess no perceivable level of low-temperature tolerance (Rizza et al., 2011; Zitzewitz et al., 2011). Spring barley is a main crop across the Canadian prairies and is used for food, malting, and general purposes (feed and forage). Spring barley yields about 20% less than winter barley, in areas where it is adapted (Kling et al., 2004). Though, spring barley genotypes are normally not as tolerant to cold as other winter cereals. Cold tolerance in barley has been a challenging phenomenon to develop with conventional breeding approaches. It is best to evaluate and screen breeding material under uniform cold stress in the laboratory as screening for winterhardiness in the field is rarely reliable. In 2021, barley-seeded areas consisted of 50.4% malting barley, 42.3% general purpose barley, and 2.3% food barley in the western Canada (Marta and Tricia, 2020). In poorer countries, barley is an essential food source (Grando and Macpherson, 2005), affording harvestable yields in locations that are harsh and marginal for crop production. Current research has categorized barley as a true functional food in more developed societies. The barley’s grain is particularly high in soluble dietary fiber, which significantly declines the risk of serious diseases such as type II diabetes. The USA Food and Drug Administration (FDA) has approved cell-wall polysaccharides from barley grain as a human health claim (Collins et al., 2010).

Selection for cold tolerance/susceptibility in barley varieties can be associated with reliable screening techniques. Traditional methods, such as measuring survival rates and plant re-growth, are time-consuming and inaccurate in quantifying the level of cold tolerance (Novillo et al., 2004). Some techniques focus on injuries to the plasma membranes that result in the leakage of electrolytes from plant tissues (Steponkus et al., 1990). The electrolyte leakage is easily quantified by conductivity measurements (Rohde et al., 2004). For such investigations, plants are exposed to freeze-thaw cycles with minimum temperatures usually ranging from -1 to -50°C, and electrolyte leakage caused by cooling is then measured. The methods to quantify the cold tolerance of plants should be non-invasive and applicable at a high-throughput screening rate so that the underlying genetic determinations can be effectively quantified and established (Mishra et al., 2011). Evidence suggests that chlorophyll fluorescence emission can be used effectively in high-throughput screening of plants’ low-temperature tolerance (Mishra et al., 2011).

Photosynthesis is one of the most essential and complex physiological mechanisms in all plants and is influenced in all periods by stresses (Baker, 1996). Because the photosynthesis mechanism regulates multiple cellular processes, including photosystems and photosynthetic pigments, electron transport system, and CO_2_ reduction events, any stress-induced damage can completely decrease the photosynthetic capacity of green plants (Lawlor and Cornic, 2002; Ashraf and Harris, 2013). Cold stress influences the photosynthetic apparatus through the suppression of photosystems and pigment modifications, thylakoid membranes, photosynthesis-related enzyme activity, chlorophyll fluorescence, gas exchange, and reduced CO_2_ assimilation in addition to the electron transport rate. Photosynthesis converts light energy into redox equivalents (NADPH) and ATP, essential elements for plant growth and development. On the other hand, cold stress prevents thylakoid electron transport *via* aggregate membrane viscosity. It disorders light energy process trapping by Photosystem I and Photosystem II antenna. The enhancement of energy trapping beyond its regular ability results in a high-energy state, which eventually leads to the overproduction of reactive oxygen species (ROS) (Ashraf and Harris, 2013).

Chlorophyll fluorescence parameters have become a common and powerful technique in plant breeding to investigate the impact of stresses on the photosynthetic mechanism (Guidi et al., 2019). It can be used as a representative of plant stress as environmental stresses, such as extremes of temperature, water, and light availability can decrease the capability of a plant to metabolize normally. This can lead to an imbalance between the light energy absorption *via* chlorophyll and the utilization of energy in photosynthesis (Schreiber, 1986; Schreiber et al., 1986). Chlorophyll fluorescence is an indicator of photosynthetic energy that responds to alterations in Photosystem II (PSII) photochemistry and consequently represents a rapid and efficient tool to assess the capacity of the photosynthetic mechanism at low temperatures (Lichtenthaler and Rinderle, 1988). The function of the photosynthetic machinery can be evaluated by measuring the ratio of chlorophyll variable fluorescence (F_V_) over the maximum fluorescence value (F_M_), which reveals the efficiency of the excitation capture by open photosystem II reaction centers (Fracheboud et al., 1999; Rizza et al., 2001). An association between the decrease of F_V_/F_M_ and frost tolerance during hardening and after freezing was found in winter wheat (Clement and Hasselt, 1996), spring and winter barleys, and rye (Smillie and Hetherington, 1983).

During cold acclimation, different physiological and biochemical modifications occur, such as the synthesizing of proline, soluble sugars, and cold-resistance proteins to maintain proteins (Hannah et al., 2010). These events play significant roles in the response to cold stress by controlling the ice crystal formation, osmotic potential, reactive oxygen species, and stability of cell walls and membranes (Ding et al., 2019). Some elements, including protein kinases, messenger molecules, phosphatases, and transcription factors (TFs), have been reported for cold-stress signaling pathways (Ding et al., 2019). Understanding the genetic regulation underlying the photosynthesis process under low-temperature stress in barley can facilitate the development of climate-resilient and high-yielding cultivars in a short period of time. Since the first draft reference of the barley genome was released (Schulte et al., 2009; IBGS et al., 2012), high‐quality genome sequences have been published (Sato et al., 2016; Mascher et al., 2017; Dai et al., 2018). These tools facilitated barley research and became precious resources for the improvement and comparative genomics studies including genome-wide association studies (GWAS) and QTL mapping. In addition, advances in genome mapping and sequencing technologies have made possible the cost-effective assembly and sequencing of hundreds of genotypes with large-genome species, such as barley (5 Gb, haploid genome size) (Monat et al., 2019; Jayakodi et al., 2020). The 9K SNP array is a commonly powerful GWAS tool for identifying specific allele variants (Comadran et al., 2012).

Evidence of chlorophyll fluorescence and photosynthesis variability in barley has been reported (Kocheva et al., 2004; Guo et al., 2007; Bertholdsson et al., 2015), indicating opportunities for genetic improvement and selection. Several analyses have been reported for quantitative trait loci (QTL) for chlorophyll fluorescence under low oxygen concentration (Bertholdsson et al., 2015), post-flowering under drought (Guo et al., 2007), early short-time drought tolerance (Wojcik-Jagla et al., 2013). Understanding the mechanisms of low-temperature tolerance and barley productivity is one of the main challenges facing scientists and breeders today. So far, the effect of low temperatures on chlorophyll fluorescence as an indicator of photosynthetic energy conversion in barley plants is still well unknown. Therefore, the aims of the present study were (i) to investigate the response of barley genotypes at three- to four-leaf stages to low-temperature stress including cold acclimation and freezing shock; (ii) to identify genetic loci associated with the chlorophyll fluorescence trait, by using genome-wide association studies; (iii) to identify key genes related to chlorophyll fluorescence before and after the LTS conditions and, (iv) introgression of cold tolerance into spring barley.

## 2 Materials and methods

### 2.1 Plant materials

A panel of 96 spring barley genotypes from eight breeding programs of Western Canadian barley breeding programs was used in this study. All genotypes were evaluated at different times between 1994 and 2006 (Beattie et al., 2010) and selected based on their high seed yield and percentage of winter survival and cold hardiness (ranging from 50 to 100%). The association panel consists of advanced breeding lines, commercial varieties, two-rowed lines used to investigate beta-glucanase and limit dextrinase, and elite germplasm, which has been developed for the Western Two-Row Cooperative Registration. Several diversity array technology (DArT) markers were detected for grain quality using 91 genotypes of this panel and disease ratings for true loose smut and net blotch (Beattie et al., 2010). In our previous study, an important pattern of genetic diversity was detected in the population studied. Our previous study revealed that this panel is differentiated due to the ear-row type and breeding program origins into five subpopulations (Capo-Chichi et al., 2023).

### 2.2 Plant growth conditions

Two experiments namely cold acclimation and freezing shock evaluations were conducted to study the impact of the low-temperature stress (LTS) on the 96 spring barley genotypes. In both experiments, ten seeds of each genotype were germinated in 8 cm×8 cm×7 cm pots containing pasteurized field soil (wet soil) in the growth chamber. The seeds in each pot were covered by an equal amount of soil to enhance uniform emergence. Pots were placed in a growth chamber at 20°C/15°C and a photoperiod of 12/12 h light/dark cycle. The experiments were designed in a completely randomized design (CRD) in three replicates. At the three-leaf seedling stage (on day 14), the germination rate was recorded for all genotypes then, seedlings were thinned to five plants per pot before the treatments.

### 2.3 Quantification of chlorophyll fluorescence

For cold acclimation treatment, seedlings at a three-leaf stage were placed in a programmable cold chamber. The initial temperature in the cold chamber was -1°C. The temperature was raised between 3°C and 5°C, then gradually decreased to -12°C over a duration of four hours (**Supplementary Figure 1**), to ensure that nucleation occurred evenly. For the freezing-shock treatment; pots were moved during the day from the growth chamber to a programmable cold chamber. The initial temperature was -18°C and the temperature was immediately raised to -6°C. Pots were exposed to temperatures that gradually declined from -6°C to -11°C for 75 min (0.06°C per min) (**Supplementary Figure 1**). In order to record chlorophyll fluorescence parameters for the same seedlings each time, the measured seedlings were numbered from 1 to 5 in both experiments. After the treatments, all seedlings were returned to the growth chambers (normal condition). A week later, a frost’s survival rate in each pot was recorded and the genotypes were characterized according to their ability to tolerate the low temperatures. Frost survival was calculated visually twice; once after the acclimation using a 1-5 symptoms scale, where: 1 (dead plant), 2 (trace of life; low survival potential), 3 (intensive damage; less than half of the coleoptile leaf green), 4 (moderate to minimal damage; limited to leaf edges), 5 (no damage).

### 2.4 Fluorescence measurements

The fluorescence measurement values were collected from 96 genotypes after cold acclimation treatment, while after the freezing-shock treatment 22 genotypes died out completely, and the results were collected from the remaining 74 genotypes. The measurements were assessed in the greenhouse on the second leaf that completely expanded. A grid with a 33 mm hole diameter was clipped on the inner section of the leaf. For a dark-adapted period, the leaf clips were left for 20 min. The measurement probe was trimmed later and the reading values were taken by using a portable chlorometer OS-30P (Opti-Sciences). Plants were allowed to dark-adapt overnight to ensure that all PSII centers are open, and the lights were turned off in the greenhouse until measurements were concluded (between 1 and 3 a.m.). The chlorophyll fluorescence parameter was measured according to the formula; (Ghassemi-Golezani et al., 2008).


FVFM=(FM− F0)/FM

Where; F_0_; F_M_ and F_V_ are primary fluorescence parameters while F_V_/F_M_ and F_V_/F_0_ are fluorescence ratios as the following.

F_0_: minimum fluorescence occurs while all antenna sites are supposed to be open (dark-adapted). F_M_: maximum fluorescence intensity under exposure to the excitation source while all antenna sites are assumed to be closed. F_V_: variable fluorescence. F_V_/F_M_: the maximum yield of primary photochemistry. F_V_/F_0_: maximum efficiency of PSII. The F_V_/F_M_ is the ratio of variable fluorescence to maximal fluorescence, which is an indicator of maximum quantum efficiency and gives important information concerning the effect of environmental stress on the plant. The F_V_/F_0_ ratio is a very sensitive indicator of the maximum efficiency of photochemical processes in PSII and/or the potential photosynthetic activity of healthy and stressed plants (Lichtenthaler & Rinderle, 1988). Chlorophyll fluorescence measurements were recorded at three different times for both experiments: before the treatment (BF), two hours after the treatment (AF), and 24 hours after the treatment (DF). Heritability in the broad sense (H_b_) was calculated before and after the treatments according to Elakhdar et al. (Elakhdar et al., 2016).

Heritability in the broad sense; $H_{b}^{2}=V_{G}^{2}/V_{P}^{2}\;\times \;100$

, where, V_G_; genetic variance and V_P_; phenotypic variance

### 2.5 DNA isolation and genotyping

Genomic DNA was isolated from the young leaf of the 3-4 leaf stage using the DNeasy Plant Mini Kit (Qiagen, Hilden, Germany). DNA quality was quantified at 230 nm and then qualified at 230/260 and 260/280 absorption ratios, respectively. Genotypes were genotyped on an Illumina 9K Barley Infinium iSelect SNP assay (Comadran et al., 2012) at the USDA-ARS genotyping laboratory (Fargo, ND). Physical positions of markers were taken from the barley pseudomolecule assembly by the James Hutton iSelect annotation (https://ics.hutton.ac.uk/50k/) and Barley DB: Barley Bioresources Database (http://earth.nig.ac.jp/~dclust/cgi-bin/index.cgi?lang=en). SNPs with unknown chromosomal position, monomorphism, and SNPs with missing values greater than 20% were eliminated. For association analysis, markers with genetic and physical positions and with minor allele frequency (MAF) of 0.05 or greater were used. After performing these filters, a total of 5063 high-quality SNP markers remained in the dataset and were used for subsequent GWAS.

### 2.6 Genome-wide association studies

Using the 5063 informative SNPs marker, a genome-wide association study (GWAS) was performed on 96 barley genotypes to identify genetic regions linked to photosynthesis after the cold. GWAS was performed using a mixed linear model (MLM) (Zhang et al., 2005), using the *R* package GAPIT (Lipka et al., 2012). The MLM model was selected due to its strength and power for detecting marker/trait associations. This model is based on the genotype data (G), population structure (Q) as fixed effects, and the Kinship-matrix (K) as random effects (Pasam et al., 2012). In our previous study, Genome-wide LD decay was plotted as R^2^ of an SNP marker against the corresponding genetic distance using the mixed-model method (Capo-Chichi et al., 2023). A strong LD with an approximate average value of 0.021 when the distance was 0.391 cM was observed among the 5063 marker pairs in the studied population. The kinship matrix was assessed using the whole set of markers. To identify significant quantitative trait nucleotides (QTNs), the Bonferroni corrected significance threshold was determined, based on the reduced marker set of 5063 SNP and a significant level of *p*<0.001 with a corresponding threshold of (-log10 p ≥ 3). Manhattan plots mapped the chromosome position on the x-axis against–log_10_ (*P*-value) on the y-axis of each marker. The quantile-quantile (QQ) was plotted between the observed and the expected -log_10_ P values.

### 2.7 Candidate gene prediction

Genes with significant markers associated with the chlorophyll fluorescence, their locations, and corresponding annotations were retrieved from the BARLEYMAP platform (http://floresta.eead.csic.es/barleymap/) (Cantalapiedra et al., 2015) version of the MorexV3 genome (Comadran et al., 2012). The physical positions of markers were revealed from the Barley Physical Map IBSC (IBGS et al., 2012), the POPSEQ map (Mascher et al., 2013), and the Morex Genome Map (Mascher et al., 2017). Gene Ontologies (GO) enrichments were obtained from the Amigo of Gene Ontology platform (http://geneontology.org) (Pomaznoy et al., 2018) and the Gene Ontology and GO annotation platform; QuickGO (https://www.ebi.ac.uk/QuickGO/). Gene co-expression for the identified genes was revealed from the Global gene co-expression networks (GCNs) database PlantNexus (Zhou et al., 2022).

### 2.8 Introgression of cold tolerance into spring barley from winter barley

Four two-rowed winter barleys ‘02Ab671’, ‘02Ab431’, ‘02Ab669’ and ‘2Ab08X05W061-208’ were obtained from the United States Department of Agriculture (USDA). The genotypes were selected based on excellent malt extract, high seed yield, and their high percentage of cold hardiness and winter survival (50 to 100% across different locations in the northern United States). Vernalization was performed in growth chambers at 5°C for 8 h of light for seedlings at the two-to-three-leaf stage. After ten weeks of vernalization, seedlings were moved to 20°C with 16/8 h light/dark, and humidity closely monitored. Upon flowering, eighteen crosses were made between spring and winter barleys (**Supplemental Table 1**).

## 3 Results

### 3.1 Germination and survival rates

**Figure 1:**
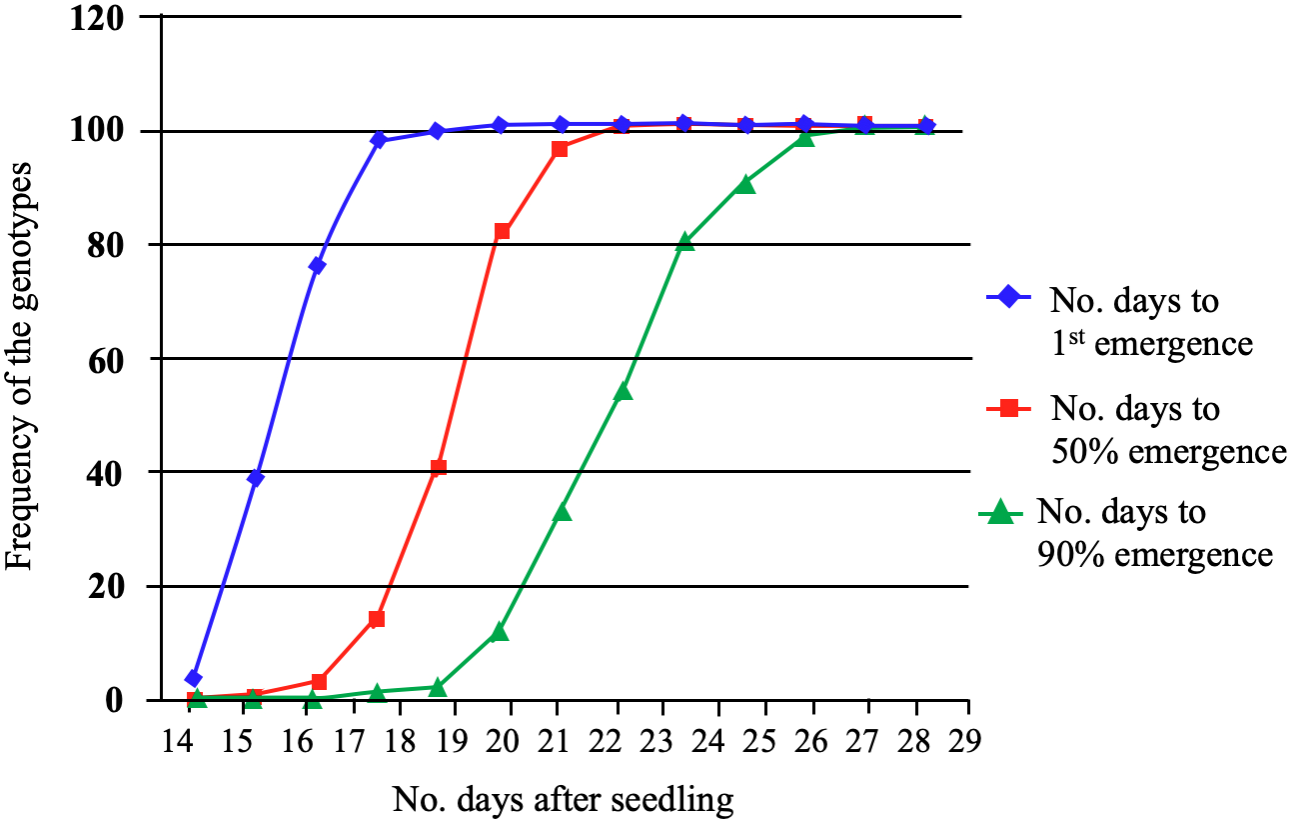
The emergence percentage (G%) of the first, 50% (T50) and greater than 90% (T>90) emergence of 96 spring barley genotypes. G% was calculated by dividing the number of emerged seedlings by the number of seeds planted for each seed plot and multiplying the product by 100.

Twenty-nine-day-old plants from 96 spring barley genotypes were used to study the impact of low-temperature stress: cold acclimation and freezing shock. The survival rates were assessed using a visual damage scale. In the cold acclimation and freeze shock experiments, the frequency of the genotypes, and distribution to germination were recorded for all genotypes (**Figure 1**). The results revealed that the time required for the emergence onset varied from 14 to 19 days for the genotypes studied (**Figure 1**). At 14 days, the emergence rates ranged from 0 to 38%, while on day 19, the emergence rates varied from 4 to 100%. The time required for 50% emergence ranged from 15 to 22 days, demonstrating that some genotypes have the ability to emerge faster than others. We observed several lines that exhibited adequate performance at 5°C.

**Figure 2:**
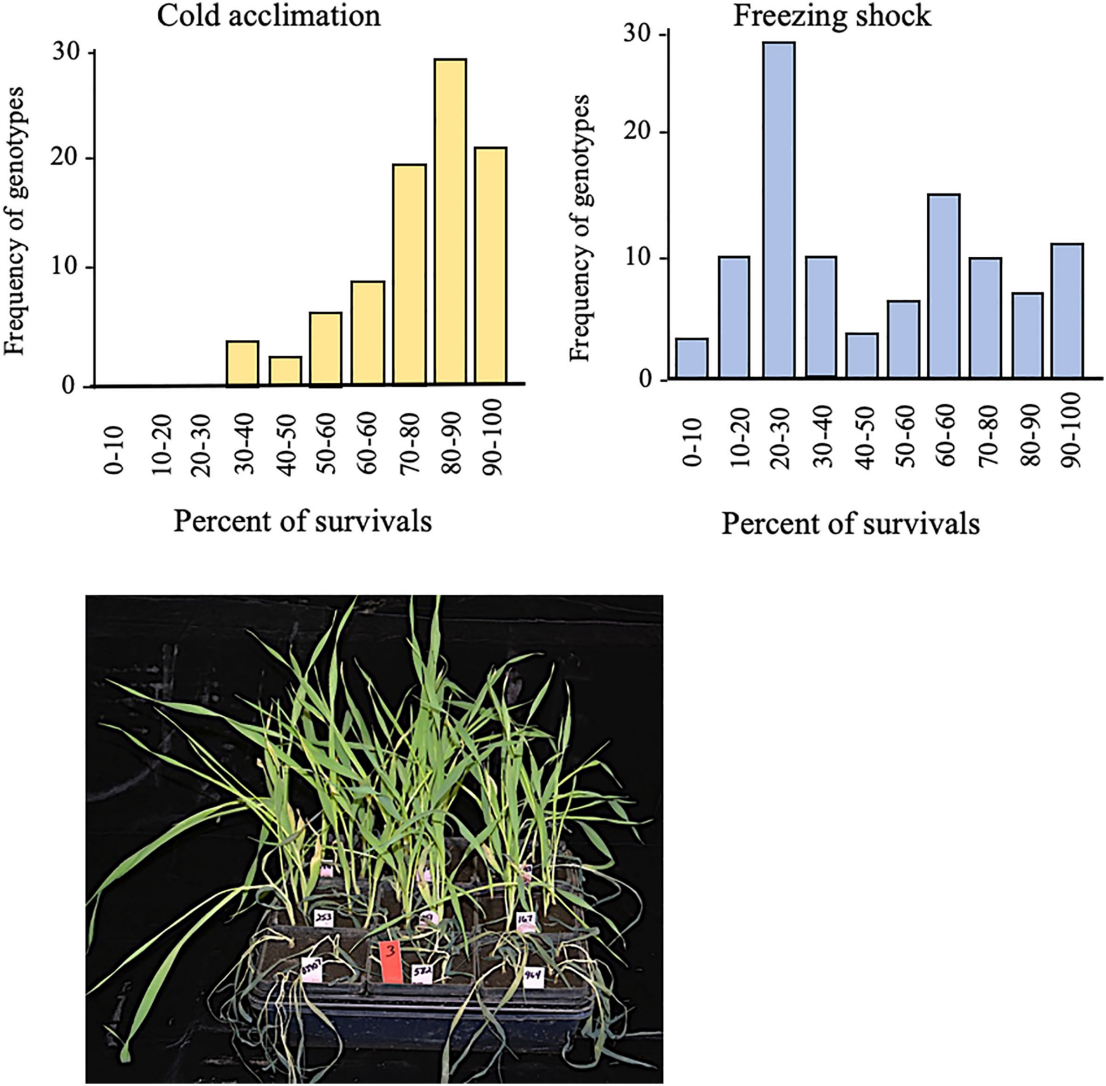
Survival rates distribution for 96 genotypes of spring barley one week old after low-temperature stress conditions. Cold acclimation (left) and freezing shock (right). View for the effect of freezing-shock treatment on the spring barley at the three-leaf stage (lower panel). Twenty-two genotypes died out completely. Low-temperature stress is induced at the three-leaf stage.

Under cold acclimation, we observed that survival frequencies ranged from 30 to 100% with the majority grouped between 80 and 90% (**Figure 2**). Under freezing-shock stress, 22 genotypes were dead while survival rates ranged from 0 to 100% with the majority of the genotypes between 20 and 30% survival (**Figure 2**). The seedlings stressed under these conditions suffered leaf wilting as the duration of stress was prolonged. These findings suggest that the cold acclimation treatment improved freezing tolerance more than the freezing shock treatment.

### 3.2 Phenotypic evaluation of chlorophyll fluorescence under LTS

**Figure 3:**
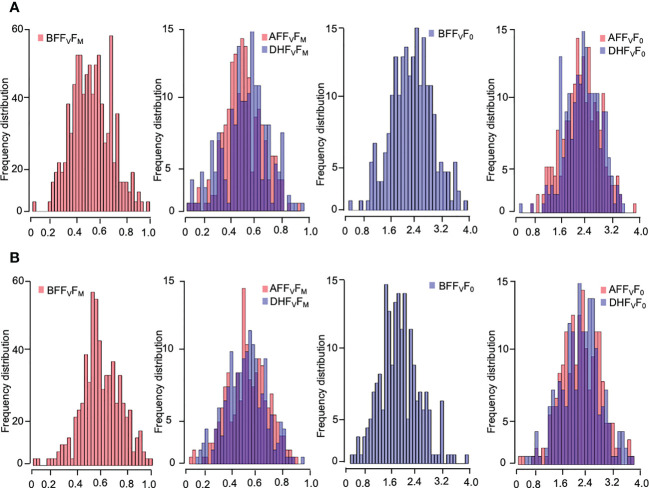
Frequency distribution of the chlorophyll fluorescence-related traits of four weeks-old barley plants under low-temperature stress; **(A)** cold acclamation and **(B)** freezing shock and overlap between them. Chlorophyll fluorescence parameters: F_V_/F_M,_ and F_V_/F_0_ values measured. Histograms with melt colors built with base R function (The R and Python graph galleries websites).

Five chlorophyll characteristics including parameters of transient fluorescence were collected for cold acclimation and freezing shock treatments. The parameters represent the photochemical efficiency of PSII behavior affected by LTS (**Table 1**). A high level of phenotypic variation was detected in all traits measured under both low-temperature stress conditions (**Table 1**). Before the treatments (BF), the highest H_b_ value was observed for F_V_/F_0_ (97.9% and 93.6% for cold acclimation and freezing shock treatments, respectively) (**Table 1**). Low to high values of heritability (H_b_) were observed under LTS treatments. Two hours after treatments (AF), F_V_/F_0_ exhibited also the highest value of heritability H_b_ of 94.4% and 96.5%, cold acclimation and freezing shock, respectively, whereas, after 24 hours of stress (DF), F_V_/F_0_ and F_M_ had the highest values 92.2% and 73.5, respectively) (**Table 1**). These results suggest adequate variability and different responses to low-temperature stress that exists in the barley material studied. Normal distribution was detected for the chlorophyll fluorescence parameters, F_V_/F_0_ and F_V_/F_M_, before and after the low-temperature conditions (**Figure 3**). This indicates that barley’s chlorophyll fluorescence traits are quantitatively inherited and controlled by multiple genes. In addition, the genotypes might vary in their photosynthetic mechanisms in response to low-temperature tolerance, which will impact the yield and ultimately the breeder. Thus, the difference in low-temperature tolerance between the genotypes provides a foundation for studying this phenomenon for GWAS and/or QTL studies.

**Table 1:** Chlorophyll characteristics-related traits under cold acclimation and freeze shock for 96 spring barley genotypes.

Treatment			Cold acclimatization				Freezing shock						
		Max	Min	Mean	SE	H^2^_b_ (%)	Max	Min		Mean	SE	H^2^_b_ (%)	
	F_0_	307	187	262.10	3.41	91.9	329.75		170.08	263.77	5.41	76.0
Before (BF)	F_V_	1003	607	842.33	11.52	93.2	1016.67		558.42	844.94	15.57	57.4
	F_M_	1308	797	1104.40	14.81	90.4	1345.33		730.17	1108.71	20.85	55.2
	F_V_/F_M_	0.775	0.746	0.76	0.00	95.1	0.78		0.75	0.76	0.00	53.9
	F_V_/F_0_	3.453	2.943	3.22	0.01	97.9	3.53		2.97	3.22	0.02	93.6
	F_0_	357	183	260.03	5.21	88.1	313.17		179.33	261.44	3.88	86.6
2 hours (AF)	F_V_	757	14	342.17	23.14	89.1	571.33		33.22	230.22	13.72	50.2
	F_M_	1086	228	602.24	27.28	90.6	851.25		258.09	491.66	15.05	46.1
	F_V_/F_M_	0.667	0.062	0.39	0.02	90.5	0.55		0.10	0.30	0.01	81.4
	F_V_/F_0_	2.513	0.067	1.28	0.08	94.4	2.00		0.12	0.86	0.05	96.5
	F_0_	295	111	209.42	5.45	97.0	270.58		127.08	194.26	4.99	72.8
	F_V_	773	1	362.66	25.10	84.2	605.25		0.33	249.71	18.41	41.6
24 hours (DH)	F_M_	1068	132	551.97	30.13	92.2	866.67		135.89	443.98	22.88	73.5
	F_V_/F_M_	0.714	0.001	0.38	0.02	88.4	0.656		0.014	0.298	0.02	72.2
	F_V_/F_0_	2.62	0.001	1.33	0.09	92.2	2.302		0.199	0.98	0.06	71.4

Max, Maximum; Min, Minimum; SE, the standard error. Broad-sense heritability (H_b_). F_0_; Minimum Fluorescence when all photosystem II (PSII) is open, F_V_=F_M_-F_0_; Variable fluorescence, F_M_; Maximum fluorescence when all PSII are closed, F_V_/F_M_; the ratio of photochemical efficiency of photosystem II (PSII), F_V_/F_0_; the ratio of maximum efficiency of photochemical processes in PSII of healthy and stressed plants.

Before the cold acclimation treatment (BF), the ratio of variable fluorescence to maximal fluorescence (F_V_/F_M_) values for lines ranged from 0.746 to 0.775, with a mean value of 0.76 (**Table 1**). The variable fluorescence to fluorescence occurring while antenna sites are supposed to be open (F_V_/F_0_) ranged from 2.943 to 3.453 with an average value of 3.22 (**Table 1**). After the exposure to low-temperature stress, the mean values of F_V_/F_M_ and F_V_/F_0_ decreased significantly in both experiments compared with those before the treatment (**Table 1**). Two hours after cold acclimation stress (AF) treatment, the F_V_/F_M_ values measured ranged from 0.062 to 0.667 with an average of 0.39 across the genotypes. The F_V_/F_0_ values ranged from 0.067 to 2.513, averaging 1.28. While the F_V_/F_M_ values after 24 hours of cold acclimation treatment (DH) varied from 0.714 to 0.001, averaging 0.38 (**Table 1**). The F_V_/F_0_ values ranged from 0.001 to 2.62, averaging 1.33 (**Table 1**). After the freezing-shock treatment, 22 genotypes died out completely (**Figure 2**). F_V_F_M_ values for genotypes ranged from 0.10 to 0.55 and 0.00 to 0.61 for AF and DH, respectively (**Table 1**). While the values measured for F_V_/F_0_ varied from 0.12 to 2.00, and from 0.00 to 2.15 for AF and DH, respectively (**Table 1**). The remaining 74 genotypes were used for the GWAS.

The result suggested that freezing-shock treatment caused a large decline in F_V_/F_M_ and F_V_/F_0_ values in the non-hardy cultivars. During the recovery in the growth chambers, we found that this effect was irreversible and contingent on a threshold. In most cases, the irreversible effect was associated with F_V_/F_M_ values below 0.220. For more hardy cultivars, the F_V_/F_M_ values varied between 0.477 and 0.609 after freezing shock treatment, while the F_V_/F_0_ ranged from 1.832 to 2.413. Taken together, these results show that the decreases in the chlorophyll fluorescence indexes might reveal a reduction of PSII efficiency and plant death.

**Figure 4:**
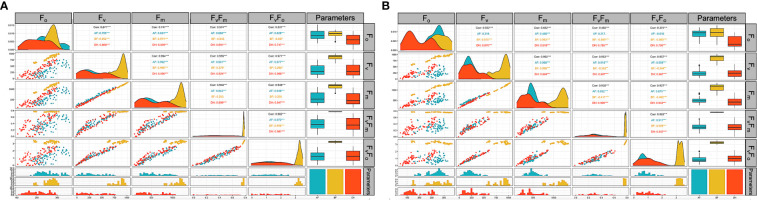
Correlation analysis of chlorophyll fluorescence parameters related traits of the three-leaf stage of 96 barley genotypes before treatment (yellow) low-temperature stress after two hours (blue) and 24 hours after the treatment (orange). Chlorophyll fluorescence parameters measured; F_0,_ F_V_, F_M_, F_V_/F_M,_ and F_V_/F_0_. **(A)**: cold acclimation treatment. **(B)**: freezing shock treatment. BF: Before treatment, AF: two hours after treatment. DH: 24 hours after the treatment. Correlation matrix built with base R function (ggally R package).

The comprehensive Pearson correlation analysis between the chlorophyll fluorescence-related traits; F_0_, F_V_, F_M_, F_V_/F_0,_ and F_V_/F_M_ under low-temperature tolerance before (BF), two hours after (AF), and 24 hours after (DH) treatment are presented in histograms (**Figure 4**). For the AF readings, correlations between the parameters measured ranged from 0.628 between F_0_ and F_V_/F_0_ to 0.992 between F_V_ and F_M_ at *p* = 0.0001. Meanwhile, a higher correlation (*p* = 0.0001) was observed under the DH stress condition between F_V_ and F_M_, which ranged from 0.691 between F_O_ and F_V_/F_M_ to 0.994 between F_V_ and F_M_ at *p* = 0.0001 (**Figure 4A**). A correlation analysis was also performed between the traits under freezing shock stress (**Figure 4B**). The correlations were moderate to high and ranged from 0.458 between F_M_ and F_0_ to 0.968 between F_M_ and F_V_ under the AF stress condition (**Figure 4** B). Under the DH condition, a positive correlation was detected between F_V_/F_0_ and F_V_/F_M_ with all studied traits and ranged from 0.755 to 0.967 when *p* = 0.0001, respectively (**Figure 4B**).

### 3.3 Genomic regions associated with chlorophyll fluorescence traits

**Figure 5:**
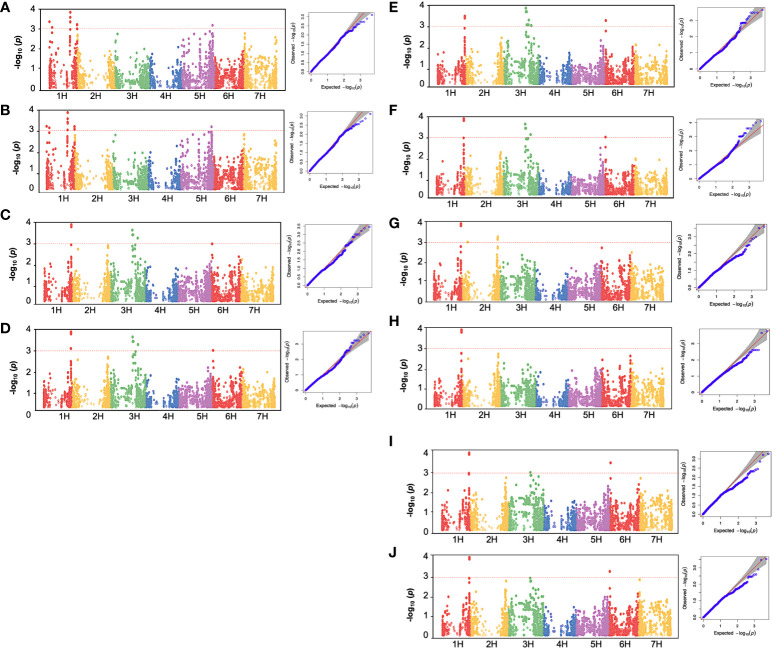
Manhattan plot and quantile-quantile (Q-Q) plot of GWAS for low-temperature stress in 96 spring barley genotypes showing significant association with chlorophyll fluorescence parameters. **(A)**: BFF_V,_ **(B)**: BFF_M,_ **(C)**: AFF_M,_ **(D)**: AFF_V,_ **(E)**: AFF_V_F_M,_ **(F)**: AFF_V_F_0_, **(G)**: DHF_M_, **(H)**: DHF_V_, **(I)**: DHF_V_F_M_, **(J)**: DHF_V_F_0_. BF: Before cold treatment, AF: two hours after cold treatment. The x-axis indicates the relative density of identified reference genome-based SNPs mapped on seven chromosomes. The y-axis represents the -log_10_ *P-*values for the association power for each SNP with the trait expressed. The peaks above-dashed lines indicate significance thresholds (- log10 *P*< 3). Barley chromosomes1H to 7H are shown.

To investigate the genetic factors associated with low-temperature tolerance in barley, a GWAS analysis was conducted for the chlorophyll fluorescence related-traits from 96 spring barley genotypes with a set of 5063 high-quality SNP markers. The average r^2^ value of the genome was 0.02, and the LD decay was found to start at an r^2^ value of 0.38 and reached half-decay at 0.2, representing the genome’s threshold distance for linkage analysis (Capo-Chichi et al., 2023). In total, 52 significant quantitative trait nucleotides (QTNs) were detected. Two significant SNPs (*P*< 0.001) were associated with chlorophyll fluorescence-related traits before low-temperature treatments, and 50 significant SNPs were observed under cold acclimation stress conditions (**Table 2**, **Figure 5**). All significant associations were found on chromosomes 1H, 3H, and 6H. While, under freezing-shock treatment, the remaining 74 cultivars used for the GWAS, the fluorescence data showed no significant associations between SNPs and chlorophyll fluorescence traits. Perhaps, this may be because of genetic drift, in which some versions of a gene have been lost because of random chance in this small population. Additionally, genetic diversity degrades more quickly in small populations than in large populations due to stochastic sampling error (genetic drift).

**Table 2:** Significant quantitative trait nucleotides (QTNs) for chlorophyll fluorescence under cold acclimation condition.

Treatment	Trait	Marker	Chr	Position (bp)^a^	*P-*value	R^2^	MAF	Allele	Effect
BF	F_M_	SCRI.RS.152795	1H	420364483	7.75E-04	0.122	0.429	A/G	67.612
	F_V_	SCRI.RS.152795	1H	420364483	7.90E-04	0.122	0.429	A/G	52.574
AF	F_M_	SCRI.RS.219551	3H	431379904	6.15E-04	0.139	0.289	T/C	128.874
		SCRI.RS.155758	1H	539454103	4.33E-04	0.149	0.378	T/C	138.895
		SCRI.RS.165588	1H	539695184	3.54E-04	0.154	0.384	A/G	144.534
	F_V_	SCRI.RS.219551	3H	431379904	3.59E-04	0.144	0.289	T/C	117.295
		BOPA1.7728.341	3H	434909031	5.94E-04	0.129	0.280	T/A	-114.505
		BOPA2.12.31368	3H	435526063	5.94E-04	0.129	0.280	A/G	-114.505
		SCRI.RS.181360	3H	440011663	5.94E-04	0.129	0.280	A/G	114.505
		SCRI.RS.138918	3H	446916578	5.94E-04	0.129	0.280	T/C	114.505
		BOPA1.ABC10084.1.2.363	3H	538660122	8.35E-04	0.120	0.179	C/G	-113.309
		SCRI.RS.144535	3H	538805589	8.35E-04	0.120	0.179	T/C	-113.309
		SCRI.RS.224360	3H	539228382	8.35E-04	0.120	0.179	T/C	113.309
		SCRI.RS.155758	1H	539454103	2.52E-04	0.154	0.378	T/C	123.775
		SCRI.RS.165588	1H	539695184	2.05E-04	0.160	0.384	A/G	128.682
	F_V_F_M_	SCRI.RS.202723	6H	13136770	8.74E-04	0.118	0.253	T/C	-0.084
		SCRI.RS.219551	3H	431379904	2.23E-04	0.157	0.289	T/C	0.094
		BOPA1.7728.341	3H	434909031	3.31E-04	0.145	0.280	T/A	-0.093
		BOPA2.12.31368	3H	435526063	3.31E-04	0.145	0.280	A/G	-0.093
		SCRI.RS.181360	3H	440011663	3.31E-04	0.145	0.280	A/G	0.093
		SCRI.RS.138918	3H	446916578	3.31E-04	0.145	0.280	T/C	0.093
		BOPA1.10126.999	3H	489991522	8.34E-04	0.119	0.295	A/G	-0.086
		SCRI.RS.155758	1H	539454103	6.70E-04	0.125	0.378	T/C	0.089
		SCRI.RS.165588	1H	539695184	5.43E-04	0.131	0.384	A/G	0.092
	F_V_F_0_	SCRI.RS.202723	6H	13136770	7.75E-04	0.118	0.253	T/C	-0.358
		SCRI.RS.219551	3H	431379904	1.53E-04	0.165	0.289	T/C	0.416
		BOPA1.7728.341	3H	434909031	2.58E-04	0.149	0.280	T/A	-0.407
		BOPA2.12.31368	3H	435526063	2.58E-04	0.149	0.280	A/G	-0.407
		SCRI.RS.181360	3H	440011663	2.58E-04	0.149	0.280	A/G	0.407
		SCRI.RS.138918	3H	446916578	2.58E-04	0.149	0.280	T/C	0.407
		BOPA1.2067.775	3H	449665313	9.70E-04	0.112	0.131	A/G	0.414
		BOPA2.12.31011	3H	462627529	9.70E-04	0.112	0.131	T/G	-0.414
		BOPA2.12.31393	3H	464907622	9.70E-04	0.112	0.131	T/C	-0.414
		BOPA1.1977.1385	3H	469771904	9.70E-04	0.112	0.131	A/G	-0.414
		BOPA1.2231.557	3H	473173134	9.70E-04	0.112	0.131	A/C	0.414
		SCRI.RS.114566	3H	482729752	9.70E-04	0.112	0.131	T/C	0.414
		BOPA1.4453.422	3H	482733343	9.70E-04	0.112	0.131	A/G	-0.414
		BOPA1.10126.999	3H	489991522	9.16E-04	0.113	0.295	A/G	-0.364
		SCRI.RS.137116	1H	536426484	8.61E-04	0.115	0.095	T/G	-0.477
		BOPA1.ABC10084.1.2.363	3H	538660122	5.66E-04	0.127	0.179	C/G	-0.386
		SCRI.RS.144535	3H	538805589	5.66E-04	0.127	0.179	T/C	-0.386
		SCRI.RS.224360	3H	539228382	5.66E-04	0.127	0.179	T/C	0.386
		SCRI.RS.155758	1H	539454103	9.83E-05	0.178	0.378	T/C	0.432
		SCRI.RS.165588	1H	539695184	7.95E-05	0.184	0.384	A/G	0.448
DH	F_M_	SCRI.RS.155758	1H	539454103	3.10E-04	0.159	0.378	T/C	157.508
		SCRI.RS.165588	1H	539695184	2.55E-04	0.164	0.384	A/G	163.702
	F_V_	SCRI.RS.155758	1H	539454103	2.19E-04	0.188	0.377	T/C	139.623
		SCRI.RS.165588	1H	539695184	1.73E-04	0.195	0.383	A/G	145.923
	F_V_F_M_	SCRI.RS.155758	1H	539454103	6.10E-04	0.144	0.378	T/C	0.114
		SCRI.RS.165588	1H	539695184	5.32E-04	0.147	0.384	A/G	0.118
	F_V_F_0_	SCRI.RS.155758	1H	539454103	3.47E-04	0.156	0.378	T/C	0.444
		SCRI.RS.165588	1H	539695184	2.95E-04	0.161	0.384	A/G	0.460

F_0_; Minimum Fluorescence when all photosystem II (PSII) is open, F_V_=F_M_-F_0_; Variable fluorescence, F_M_; Maximum fluorescence when all PSII are closed, F_V_/F_M_; the ratio of photochemical efficiency of photosystem II (PSII), F_V_/F_0_; the ratio of maximum efficiency of photochemical processes in PSII of healthy and stressed plants.

BF; before the treatment AF; 2hr after the treatment. DH; 24hr after the treatment. Ch; chromosome number. ^a^ physical position of the markers based on (Mascher et al., 2017). MAF, minor allele frequency. Effect; additive effect. *Putative QTL that may be associated with multiple traits.

Before the low-temperature stress treatment, one significant MTA was identified for F_M_ and F_V_, which is located on chromosome 1H (42.035-42.036 cM) (**Table 2**). The peak marker was SCRI.RS.152795 with likelihood ratio-based R^2^ of 1.22 and explained 67.612 and 52.574% of the additive variances for F_M_ and F_V_, respectively. The Manhattan and Q-Q plot for both F_M_ and F_V_ are shown in **Figures 5A, B**, respectively .

**Figure 6:**
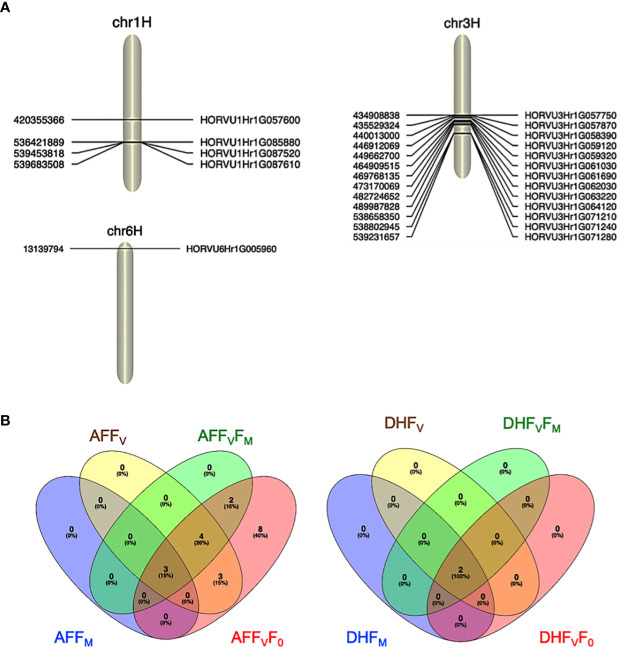
**(A)**. Significant marker associated with cold tolerance on chromosomes 1H, 3H, and 6H. **(B)** Venn diagrams showing the significant SNPs identified for all the chlorophyll photosynthetic traits across experiments; before and after the cold acclimation stress. BF, Before cold treatment; AF, two hours after cold treatment. Chlorophyll fluorescence parameters: F_0,_ F_V_, F_M_, F_V_/F_M,_ and F_V_/F_0_ values measured.

For the AF measurements under stress conditions, 39 significant QTNs were detected, three QTNs for F_M_, ten QTNs for F_V_, nine QTNs for F_V_F_M,_ and twenty for F_V_F_0_ (**Table 2**). Several QTNs were denoted in multiple chlorophyll fluorescence-related traits under both BF and/or AF conditions (**Figure 6**). There were reasonable correlations detected between chlorophyll fluorescence-related traits (**Figure 4A**) and visual damage showing that some loci contributed to both phenotypes (i.e., cold acclimation damage/photosynthetic efficiency and tolerance). Among the significantly associated markers, four SNPs were detected for F_V_, F_V_F_0,_ and F_V_F_M_ (**Table 3** and (**Figures 5D, E**). For example, BOPA1.7728.341, BOPA2.12.31368, BOPA2.12.31368, SCRI.RS.181360, and SCRI.RS.13891 have founded in the interval region on chromosome 3H between 434909031 bp and 446916578 bp (**Figure 5D**). Further, five QTNs were associated with AFF_V_, AFF_V_/F_0._ Of them, BOPA1.ABC10084.1.2.363, SCRI.RS.144535, and SCRI.RS.224360 were located between 538658350 bp and 539231657 bp in the interval region on chromosome 3H (**Table 3**). These results suggested that the QTNs identified in several traits could be considered to be more stable. For the DH readings, eight significant SNPs were detected for the chlorophyll fluorescence-related traits F_M,_ F_V_, F_V_/F_M_, and F_V_/F_0_ (**Table 3** and **Figures 5G–J**). All the above-mentioned related traits were associated with two markers SCRI.RS.155758 and SCRI.RS.165588, which were detected on chromosome 1H at the interval regions of 539454103 bp and 539695184 bp (**Figure 6**). The R^2^ values ranged between 0.112 and 0.195 for the 52 SNP markers across different traits, suggesting the presence of major QTNs controlling different chlorophyll fluorescence parameters.

**Table 3:** Functional annotation of the candidate genes associated with chlorophyll fluorescence under cold acclimation conditions.

Marker	Trait	Chr.	Marker position (bp)	Gene ID^a^	Gene class^b^	start (bp)	end	Annotation
SCRI.RS.152795	BFF_M_	1H	420364483	HORVU1Hr1G057600	HC_G	420355366	420364697	PAX-interacting protein 1
SCRI.RS.219551	AFF_M_	3H	431379904					
SCRI.RS.155758	AFF_M_, DHF_M_, DHF_V_, DHF_V_/F_M_, DHF_V_/F_0_	1H	539454103	HORVU1Hr1G087520	HC_G	539453818	539456544	E3 SUMO-protein ligase MMS21
SCRI.RS.165588	AFF_M_, DHF_M_, DHF_V_, DHF_V_/F_M_, DHF_V_/F_0_	1H	539695184	HORVU1Hr1G087610	LC_U	539683508	539700690	unknown function
BOPA1.7728.341	AFF_V_, AFF_V_/F_M_, AFF_V_/F_0_	3H	434909031	HORVU3Hr1G057750	HC_U	434908838	434910472	unknown protein
BOPA2.12.31368	AFF_V_, AFF_V_/F_M_, AFF_V_/F_0_	3H	435526063	HORVU3Hr1G057870	HC_G	435529324	435531123	Mannan endo-1,4-beta-mannosidase 7
SCRI.RS.181360	AFF_V_, AFF_V_/F_M_, AFF_V_/F_0_	3H	440011663	HORVU3Hr1G058390	HC_G	440013000	440017152	Bromodomain-containing factor 1
SCRI.RS.138918	AFF_V_, AFF_V_/F_M_, AFF_V_/F_0_	3H	446916578	HORVU3Hr1G059120	HC_U	446912069	446922626	Chromosome 3B, genomic scaffold, cultivar Chinese Spring
BOPA1.ABC10084.1.2.363	AFF_V_, AFF_V_/F_0_	3H	538660122	HORVU3Hr1G071210	HC_G	538658350	538660659	Mannan endo-1,4-beta-mannosidase 2
SCRI.RS.144535	AFF_V_, AFF_V_/F_0_	3H	538805589	HORVU3Hr1G071240	HC_G	538802945	538807675	Protein kinase superfamily protein
SCRI.RS.224360	AFF_V_, AFF_V_/F_0_	3H	539228382	HORVU3Hr1G071280	HC_u	539231657	539232192	undescribed protein
SCRI.RS.202723	AFF_V_, AFF_V_/F_0_	6H	13136770	HORVU6Hr1G005960	HC_G	13139794	13140854	histone H2A 7
BOPA1.10126.999	AFF_V_, AFF_V_/F_0_	3H	489991522	HORVU3Hr1G064120	HC_G	489987828	490006022	Alpha/beta hydrolase domain-containing protein 13
BOPA1.2067.775	AFF_V_F_0_	3H	449665313	HORVU3Hr1G059320	HC_G	449662700	449666771	V-type proton ATPase subunit E
BOPA2.12.31011	AFF_V_F_0_	3H	462627529					
BOPA2.12.31393	AFF_V_F_0_	3H	464907622	HORVU3Hr1G061030	LC_u	464909515	464910032	undescribed protein
BOPA1.1977.1385	AFF_V_F_0_	3H	469771904	HORVU3Hr1G061690	HC_G	469768135	469772099	Protein DEHYDRATION-INDUCED 19 homolog 3
BOPA1.2231.557	AFF_V_F_0_	3H	473173134	HORVU3Hr1G062030	HC_G	473170069	473175037	ROP guanine nucleotide exchange factor 5
SCRI.RS.114566	AFF_V_F_0_	3H	482729752	HORVU3Hr1G063220	HC_G	482724652	482734251	Splicing factor 3A subunit 3
BOPA1.4453.422	AFF_V_F_0_	3H	482733343					
SCRI.RS.137116	AFF_V_F_0_	1H	536426484	HORVU1Hr1G085880	HC_G	536421889	536430726	Ninja-family protein

F_0_; Minimum Fluorescence when all photosystem II (PSII) is open, F_V_=F_M_-F_0_; Variable fluorescence, F_M_; Maximum fluorescence when all PSII are closed, F_V_/F_M_; ratio of photochemical efficiency of photosystem II (PSII), F_V_/F_0_; the ratio of maximum efficiency of photochemical processes in PSII of healthy and stressed plants. BF; before the treatment. AF; 2hr after the treatment. DH; 24hr after the treatment.

a The candidate genes and their corresponding annotations were obtained from BARLEYMAP (Cantalapiedra et al., 2015). http://floresta.eead.csic.es/barleymap/.

b HC_G; high-confidence gene with predicted function, HC_U, high-confidence gene without predicted function, LC_u, low-confidence gene without predicted function (MorexV3 2021 edition).

### 3.4 Prediction of candidate genes associated with significant SNPs

The genomic regions close to the associated SNPs detected in the GWAS were annotated using BARLEYMAP, and then the gene ontologies (GO) enrichments were performed by using Amigo (**Tables 3** and 4). Of the 52 candidate genes identified by the GWAS dataset, we found that 15 genes were common among the different traits most of them with high confidence and predicted function (HC_G).

In the interval detected region between 420355366 bp and 420364697 bp on chromosome 1H, there is one gene (*HORVU1Hr1G057600*) associated with the F_M_ trait and encoding to PAX-interacting protein 1 (PAXIP1) (**Tables 3**). The PAXIP1 is involved in transcriptional regulation by histone methyltransferase (HMT) complexes and the DNA damage response. AF genes directly participate in the DNA repair pathway, post-translational modifications, and protein kinase which plays an important role in DNA replication and repair, transcription regulation, and chromosomal stability.

In the identified regions on chromosome 1H, between 539695184 bp to 536426484 bp, two common AF and DH genes (*HORVU1Hr1G087520*) belong to the E3 SUMO-protein ligase family which is involved in a DNA repair pathway. In addition, one gene has an unknown function. *HORVU1Hr1G085880* is another AF gene that encodes a ninja-family protein that plays a role in stress-related and growth-related signaling cascades (**Table 3**).

In the interval detected on chromosome 3H between 431379904 bp and 539228382 bp, there are six AF genes with undescribed protein annotations and ten annotated genes (**Table 3**). Of the ten genes, two (*HORVU3Hr1G057870* and *HORVU3Hr1G071210*) belong to the same enzyme family Mannan endo-1,4-beta-mannosidase 7 and Mannan endo-1,4-beta-mannosidase 2 protein, respectively. In addition to an enzyme (V-type proton ATPase subunit E), transcription factor (Bromodomain-containing factor 1), binding protein (Protein DEHYDRATION-INDUCED 19 homolog 3), transporter (ROP guanine nucleotide exchange factor 5) and splicing factor (splicing factor 3A subunit 3) (**Table 3**). The remaining genes are an Alpha/beta hydrolase domain-containing protein 13 and protein kinase superfamily protein.

In the interval region on chromosome 6H between 13139794 bp and 13140854 bp, one gene (*HORVU6Hr1G005960*) encodes histone H2A 7 protein, a core element of the nucleosome, which plays an important role in transcription regulation, DNA repair and replication and chromosomal stability (**Table 3**).

To provide more insight into the candidate gene products in the pathways related to low-temperature tolerance, three independent categories of gene ontology were categorized: cellular components (CC), biological processes (BP), and molecular functions (MF) were categorized. The discovered candidate genes were annotated, and several GO terms were observed to be mainly relevant to low-temperature tolerance and chlorophyll fluorescence (**Table 4**). We identified 16 GO terms using the discovered genes. We found that four unique GO terms, including the zinc ion binding term (GO:0008270), hydrolase activity (GO:0004553), carbohydrate metabolic process (GO:0005975), nuclear-transcribed mRNA catabolic process, no-go mRNA decay (GO:0005634) exhibited an overrepresentation of candidate genes linked with chlorophyll fluorescence-related traits under cold acclimation stress, AF and DH treatments. Interstitially, two GO terms GO:0005515 and GO:0005634 presented no-go mRNA decay as one of the mRNA surveillance pathways. We suggested that most of the identified genes might be directly and/or indirectly involved in the photosynthetic energy conversion in barley plants under low-temperature stress responses.

**Table 4:** Gene Ontology annotation of the candidate genes on located on chromosomes 1H, 3H and 6H and their respective functional annotations.

Gene ID	InterPro	Gene Ontologies	Term	Definition	Synonyms		PFAM
HORVU1Hr1G057600	IPR001357 IPR016181						PF00533
HORVU1Hr1G087520	IPR004181 IPR013083	GO:0008270	zinc ion binding	Binding to a zinc ion (Zn).	Zn binding	MF	PF11789
HORVU1Hr1G087610							
HORVU3Hr1G057750							
HORVU3Hr1G057870	IPR013781 IPR017853 IPR018087	GO:0004553	hydrolase activity, hydrolyzing O-glycosyl compounds	Catalysis of the hydrolysis of any O-glycosyl bond.	O-glucosyl hydrolase activity	MF	
		GO:0005975	carbohydrate metabolic process	The chemical reactions and pathways involving carbohydrates, any of a group of organic compounds based of the general formula Cx (H_2_O) y	carbohydrate metabolism	BP	
HORVU3Hr1G058390	IPR001487	GO:0005515	nuclear-transcribed mRNA catabolic process, no-go decay	The chemical reactions and pathways resulting in the breakdown of the transcript body of a nuclear-transcribed mRNA with stalls in translation elongation.	no-go decay/no-go mRNA decay	BP	PF00439
HORVU3Hr1G059120							
HORVU3Hr1G071210	IPR018087 IPR001547 IPR013781 IPR017853	GO:0004553	hydrolase activity, hydrolyzing O-glycosyl compounds	Catalysis of the hydrolysis of any O-glycosyl bond.	O-glucosyl hydrolase activity	MF	PF00150
		GO:0005975	carbohydrate metabolic process	The chemical reactions and pathways involving carbohydrates, any of a group of organic compounds based of the general formula Cx(H_2_O)y	carbohydrate metabolism	BP	
HORVU3Hr1G071240	IPR008271 IPR011009 IPR000719 IPR001245 IPR002290	GO:0004672	protein kinase activity	Catalysis of the phosphorylation of an amino acid residue in a protein, usually according to the reaction: a protein + ATP = a phosphoprotein + ADP.	protamine kinase activity	MF	PF07714
		GO:0005524	ATP binding	Binding to ATP, adenosine 5’-triphosphate, a universally important coenzyme and enzyme regulator.		MF	
		GO:0006468	protein phosphorylation	The process of introducing a phosphate group on to a protein.	protein amino acid phosphorylation	BP	
HORVU3Hr1G071280							
HORVU6Hr1G005960	IPR032454 IPR032458 IPR002119 IPR007125 IPR009072	GO:0046982	protein heterodimerization activity	Binding to a nonidentical protein to form a heterodimer.		MF	PF00125 PF16211
		GO:0000786	nucleosome	A complex comprised of DNA wound around a multisubunit core and associated proteins, which forms the primary packing unit of DNA into higher order structures	cytoplasmic nucleosome/nuclear nucleosome	CC	
		GO:0003677	DNA binding	Any molecular function by which a gene product interacts selectively and non-covalently with DNA (deoxyribonucleic acid).	microtubule/chromatin interaction structure specific DNA binding plasmid binding	MF	
		GO:0005634	nuclear-transcribed mRNA catabolic process, no-go decay	The chemical reactions and pathways resulting in the breakdown of the transcript body of a nuclear-transcribed mRNA with stalls in translation elongation	no-go decay no-go mRNA decay	BP	
HORVU3Hr1G064120	IPR029058 IPR029059						PF12695
HORVU3Hr1G059320	IPR002842	GO:0015991	proton transmembrane transport	The directed movement of a proton across a membrane.	hydrogen ion transmembrane transport	BP	PF01991
		GO:0033178	proton-transporting two-sector ATPase complex, catalytic domain	A protein complex that forms part of a proton-transporting two-sector ATPase complex and catalyzes ATP hydrolysis or synthesis. The catalytic domain (F1, V1, or A1) comprises a hexameric catalytic core and a central stalk, and is peripherally associated with the membrane when the two-sector ATPase is assembled.		CC	
		GO:0046961	proton-transporting ATPase activity, rotational mechanism	Enables the transfer of protons from one side of a membrane to the other according to the reaction: ATP + H2O + H+(in) = ADP + phosphate + H+(out), by a rotational mechanism.	ATP synthase activity	MF	
HORVU3Hr1G061030							
HORVU3Hr1G061690	IPR008598 IPR027935						PF05605 PF14571
HORVU3Hr1G062030	IPR005512	GO:0005089	guanyl-nucleotide exchange factor activity	Stimulates the exchange of GDP to GTP on a signaling GTPase, changing its conformation to its active form. Guanine nucleotide exchange factors (GEFs) act by stimulating the release of guanosine diphosphate (GDP) to allow binding of guanosine triphosphate (GTP), which is more abundant in the cell under normal cellular physiological conditions.	GDP-dissociation stimulator activity	MF	PF03759
HORVU3Hr1G063220	IPR031774 IPR000690 IPR021966 IPR024598	GO:0003676	nucleic acid binding	Binding to a nucleic acid.	base pairing	MF	PF16837 PF11931 PF12108 PF13297
		GO:0005634	nuclear-transcribed mRNA catabolic process, no-go decay	The chemical reactions and pathways resulting in the breakdown of the transcript body of a nuclear-transcribed mRNA with stalls in translation elongation.	no-go decay	BP	
		GO:0008270	zinc ion binding	Binding to a zinc ion (Zn).	Zn binding	MF	
HORVU1Hr1G085880	IPR012463 IPR032308						PF07897 PF16135

MF, Molecular Function; BP, Biological Process; CC, Cellular components.

### 3.5 Cold tolerance introgression into spring barley

**Figure 7:**
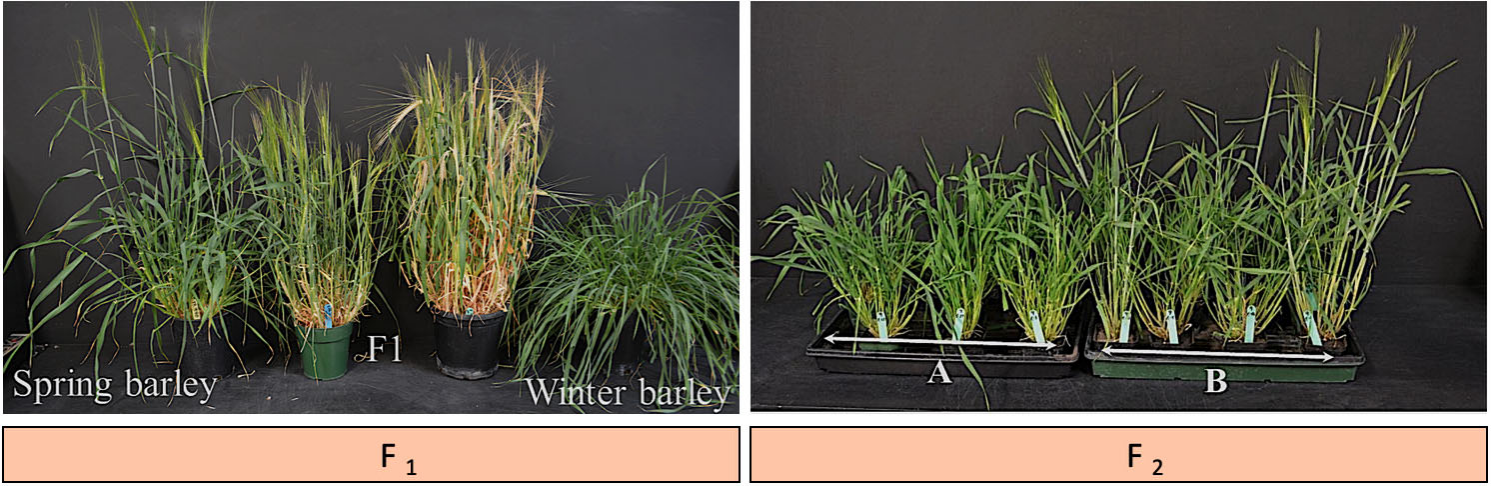
Prospects for Growth Habit. Two F_1_ populations were derived from a cross between 02Ab431 (♀- winter type) and Bentley (♂- spring type). F_2_ population segregated for growth habit (spring versus winter type). The photo was taken 39 days after seeding. **(A)**: genotypes showing winter type. **(B)**: genotypes showing spring type with early flowering.

To develop new low temperature-tolerant varieties/germplasm of spring barley as a part of the current project, F_1_ populations were produced from the cross-between winter and spring barleys. A single seed of each of the F_1_ populations was planted per pot and growth habits were recorded. The results showed that winter barleys exhibit no flowers while all tillers of spring barleys exhibit fully flowered (**Figure 7**). We observed the number of tillers is high in F_1_ populations in comparison with the spring type of the parental lines. F_2_ populations were segregated for growth habits (**Figure 7**). The genotypes with spring growth habits were selected and evaluated for cold tolerance. We identified some spring F_2_ plants earlier to flowering than the parental lines. These genotypes entered advanced generations (RIL_2-5_) and were validated in field conditions to be used in the breeding programs towards the breeding of high and stable-yielding varieties in the Canadian prairies. We, therefore, suggest additional studies to refine and validate the cold hardiness of these lines such as QTL mapping to identify and characterize the candidate genes underlying this quantitative trait. Together, this study demonstrated that cold hardiness can be introgressed into spring barleys from winter barleys easily without the restriction of genes transferring related to vernalization requirement.

## 4 Discussion

Identifying and characterizing key genes underlying low-temperature tolerance has become the main priority for improving the hardiness of barley. A deeper comprehension of the regulation networking and pathways of these genes and their association with low-temperature stress (LTS) would assist in the illustration of how barley plants adapt to stress. Understanding these pathways might offer opportunities for increasing the levels of cold tolerance. Due to the complexity of injuries and symptoms, it is difficult to measure the cold tolerance of barley at the seedling stage. This study evaluated cold tolerance in barley using visual symptoms and chlorophyll fluorescence as indicators of photosynthetic energy conversion.

### 4.1 Natural variation in chlorophyll fluorescence traits

Under cold acclimation and freezing shock stresses, the F_V_F_M_ and F_V_/F_0_ declined rapidly as an index of freezing tolerance and subsequent loss of viability (**Table 1**). We found that the time for full leaf wilting was shorter with the freezing shock treatment compared with the cold acclimation treatment. Evidence suggested that wilting was mainly initiated by reduced water- uptake, and the stomatal response to water stress was not influenced by lowered temperature (Benson, 2008). The reduction in osmotic potential accounted for an enhancement in sugars. Sugars are believed to function principally as cryoprotectants (Benson, 2008). The accumulation of these solutes at low temperatures is essential not only for freezing tolerance but also for the prevention of cell dehydration (Wang M. et al., 2021). Though other photosynthetic parameters might be used as indicators of viability, the F_V_F_M_ parameter is suggested for some reasons. First, a minor change in the F_V_F_M_ value is easily visible and indicates clearly that loss of viability is imminent. The consistency of the F_V_F_M_ parameter also increases the ease with which a threshold level can be defined. More significantly, dissimilar light-dependent parameters, F_V_F_M_ is obtained from specimens in the dark-adapted state, negating the need for an extended period of illumination before measurement. As a result, because the quantity of F_V_F_M_ can be performed with a single saturating pulse, a rapid evaluation of a large number of plants can be evaluated quickly in a short time.

An association between the decrease of F_V_/F_M_ and frost tolerance during hardening and after freezing was observed in winter wheat (Clement and Hasselt, 1996). F_V_/F_M_ values showed a significantly decreased in studied genotypes of oat (*Avena sativa* L.) during acclimation to low, nonfreezing temperatures. F_V_/F_M_ measurement was also found to be highly associated with frost damage evaluation under field conditions (Rizza et al., 2001). The decreases in the chlorophyll fluorescence indexes in this study are revealing the reduction in PSII efficiency. This could lead to a decline in photosynthetic activity mainly because of a light-induced decrease in CO_2_ assimilation and accumulation of reactive oxygen species (ROS), which, in turn, prevents protein synthesis (Baker, 1996). Even though the decrease in photo-assimilation could be based on the damage to various elements of the photosynthetic apparatus, the term photoinhibition is commonly used to describe light-induced inhibition of the PSII activity (Murata et al., 2007). The produced ROS are accountable for the damage to PSII reaction centers by inhibiting the protein synthesis, which is required for the PSII repair, resulting in the stimulation of PSII photoinhibition (Murata et al., 2007). PSII is the most prone component to be destroyed in the thylakoid membranes. Hence, the main result of abiotic stress is to make PSII susceptible to photoinhibition. In this study, the association mapping materials were selected based on grain yield and a high percentage of winter survival. We found significant phenotypic variation among cultivars for chlorophyll fluorescence-related traits, including F_0_, F_V_, F_M_, F_V_/F_0,_ and F_V_/F_M_ before (BF), two hours (AF), and 24 hours (DH) after low-temperature treatment. These outcomes suggest that this panel could be used as a genetic source of seedling low-temperature tolerance in Western Canadian barley breeding programs.

### 4.2 GWAS analysis provided potential SNP markers for chlorophyll fluorescence traits

The chlorophyll fluorescence traits characterized in this study are quantitative traits, thus, several genomic regions with small effects that contribute to the phenotype are expected. We first identified the genes having significant SNPs (**Tables 2**, **3**) and discovered several candidate genes underlying chlorophyll fluorescence trait variation. Gene expression for some identified genes in different tissues of the barley plant was revealed from the Global gene co-expression networks (GCNs) database (**Supplementary Figure 2**).

Among these, 13 genes are associated with the chlorophyll fluorescence trait under cold acclimation stress. Interestingly, we found two SNP markers on chromosome 1H that have been reported to be correlated with environmental stress. On chromosome 1H, a peak SNP was discovered to be associated with AFF_M_, DHF_M_, DHF_V_, DHF_V_/F_M_, and DHF_V_/F_0_ that encode E3 SUMO-protein ligase. SUMO is a small ubiquitin-related modifier conjugation that is essential for posttranslational modification controlled by environmental cues (Miura et al., 2007a; Miura and Hasegawa, 2010). SUMO conjugation/deconjugation was involved in responses to oxidative stress, heat shock, phosphate limitation, hypoxia, flowering, pathogen defense, and ABA signaling (Kurepa et al., 2003; Lois et al., 2003; Murtas et al., 2003; Miura et al., 2005; Yoo et al., 2006; Lee et al., 2007). In *Arabidopsis* AtSIZ1 SUMO E3 ligase functions are conserved in several environmental responses including cold tolerance responses, salicylic acid in plant defense, basal thermotolerance, flowering time regulation, and freezing tolerance (Miura et al., 2005; Yoo et al., 2006; Lee et al., 2007; Miura et al., 2007b). Overexpression of the rice *OsSIZ1* gene improved tolerance to drought, heat and salt tolerance in Arabidopsis (Mishra et al., 2018), and drought and heat tolerance in cotton (Mishra et al., 2017). Furthermore, they produced higher seed yields under different stress conditions and improved the photosynthesis rate of plants exposed to heat stress. The SUMO E3 Ligase SIZ1 is involved in anthocyanin accumulation under high light in *Arabidopsi*s (Zheng et al., 2020), DNA demethylation (Kong et al., 2020), numerous abiotic stresses (Fang et al., 2022), phosphate homeostasis in rice (Pei et al., 2020), and protecting maize plants from paraquat toxicity (Wang H.et al., 2021). The *siz1* mutation exhibits freezing sensitivity as a decrease in expression of *CBF3/DREB1*, a transcription factor for acclimation to cold temperatures (Miura and Hasegawa, 2010). Recently, the *HORVU3Hr1G016010* gene encodes a SUMO found to be involved in the cold response in the VIR barley collection (2214 accession) (Sallam et al., 2021). In this investigation, we found that the *HORVU1Hr1G087520* on chromosome 1H encodes E3 SUMO-protein ligase associated with the chlorophyll fluorescence trait under low-temperature conditions and could regulate the photosynthesis rate. Photosynthetic proteins are responsible for providing the cells with the energy to assemble the defensive molecules in response to stress (Ashraf and Harris, 2013). Thus, E3 SUMO could be essential for cold acclimation tolerances in barley at the posttranslational modification level.

Further, we found a significant SNP associated with AFF_V_F_0_ under cold acclimation stress in a gene *HORVU1Hr1G085880* encoding Ninja-family protein. Ninja (NOVEL-INTERACTOR-OF-JAZ) protein regulates growth-related and stress-related signaling (Pauwels et al., 2010). ECAP NINJA is an adaptor protein linking JASMONATE-ZIM DOMAIN (JAZ) proteins with the co-repressor, TOPLESS (TPL), to regulate gene suppression in Jasmonate (JA)-dependent the *Arabidopsis* root growth inhibition. ECAP interacts with JAZ6 and JAZ8, as an adaptor protein to inhibit the JA-responsive anthocyanin accumulation by TPR2 recruitment to the transcriptional complex (Li et al., 2020). Through protein Ninja, Jasmonate JAZ repressor proteins recruit the Groucho/Tup1-type co-repressor TPL and TPL-related proteins (TPRs) (Pauwels et al., 2010). In *Arabidopsis thaliana*, Ninja binding proteins AFP1 and AFP4 are key regulators of ABA signaling and stress responses in seedlings, as well as a negative regulator of jasmonate responses (Garcia et al., 2008). NINJA, the novel interaction of JAZ encodes a transcriptional repressor that participated in the jasmonic acid pathway in the leaf and root growth and development (Oblessuc et al., 2020). Phytohormones such as JA, ABA, and salicylic acid are essential elements of complex signaling networks of the stress response (Lee et al., 2007) and control gene expression.

Based on our results, we have proven that the post-translational modification of protein related to photosynthesis is critical for barley plants, which dynamically modulated their response to environmental changes under LTS conditions. We also reported that SUMO E3 ligase could accelerate the ubiquitin-proteasome system, by conjugation of small ubiquitin-like modifiers to target substrate proteins (cold tolerance-related genes) (Johnson, 2004). Ubiquitin could likewise alter some key TFs that facilitate plant adaptation to cold stress (Callis, 2014). Our results revealed that GWAS identified two SNPs on chromosomes 1H located between 539454103 bp (SCRI.RS.155758) and 536426484 bp (SCRI.RS.137116) linked to the E3 SUMO and Ninja proteins, respectively (**Table 3**).

Interestingly, after two hours of the cold acclimation stress, we discovered SNPs on chromosome 3H, detected at 440011663 bp within the *HORVU3Hr1G058390* gene encoding a Bromodomain-containing factor 1 protein was found to be associated with the F_V_, F_V_/F_M,_ and F_V_/F_0_. Bromodomain-containing transcription factors control gene expression in three ways; (i) transcription activation, (ii) conserving transcription memory, and (ii) anti-silencing of the conserved chromosomal region (Loyola and Almouzni, 2004). The bromodomain proteins proceed with these functions *via* binding to acetylated histones and anchoring sequence-specific factors on the chromatin of target promoters. Further, bromodomain-containing proteins contain various families of ‘epigenetic mark readers’, which are basic to epigenetic gene regulation, and necessary for different environmental stress responses and cellular processes (Abiraami et al., 2022). In Arabidopsis, the bromodomain-containing transcription factors, GTE9, and GTE11 convert specific factors as BT2 to control gene expression. BT2 is a BTB domain-containing protein with an essential TAZ-zinc finger domain and calmodulin-binding domain C-terminal that responds to different metabolic and physiological responses (Mandadi et al., 2009). Loss-of-function mutants *bt2* exhibited a hypersensitive response to ABA- and sugar-mediated suppression of germination, suggesting the role of ABA in sugar signaling in germination and development. *BT2* expression was controlled by several abiotic and biotic stresses including cold, ABA, hydrogen peroxide (H_2_O_2_), and methyl jasmonate (Mandadi et al., 2009). Cold-stress signal perception is the first stage, which is completed by various pathways. Transcriptional regulations are the next factors that control ABA-dependent signaling pathways to prompt the expression of cold-regulated genes. Resulting in upregulating of hundreds of metabolite levels, some of which are identified to have protective results against the negative impacts of cold stress including soluble sugars, ROS, and photosynthetic metabolites (Heidarvand and Maali Amiri, 2010).

On the other hand, we found SNPs on chromosome 6H located within the *HORVU6Hr1G005960* gene that encodes histone H2A 7 protein, which has been associated with multiple traits, including F_V_, F_V_/F_0_ under the cold acclimation condition. Histone H2A is one of the five key histone proteins engaged in the chromatin structure in eukaryotic cells that are responsible for maintaining the shape and structure of a nucleosome. Histone modification is an epigenetic mechanism involving changes in the chromatin structure of stressed genes *via* several chemical processes during the transcriptional and post-transcriptional modifications (Kim et al., 2015). These mechanisms play an essential role in plant survival under adverse environmental conditions (Elakhdar et al., 2022; Verma et al., 2022). In addition, epigenetic mechanisms might create stress memory which is convenient for the following generations (Verma et al., 2022), to regulate gene expression to cope with environmental stresses. Histone genes are suppressed by abiotic stresses such as drought (Kang et al., 2011), cold (Steward et al., 2000), and chilling tolerance (Zhang et al., 2012).

An SNP located at 538805589 bp within the *HORVU3Hr1G071240* gene on chromosome 3H associated with F_V_, F_V_/F_0_ after two hours of the cold acclimation stress falls within protein kinase superfamily protein. Protein kinases belonging to a wide superfamily play an essential role in numerous biological processes of plant growth and stress tolerance. In parallel, several stress-inducible protein kinase families for instance calcium-dependent protein kinase (CDPK), mitogen-activated protein kinase (MAPK), and SNF1-related protein kinase (SnRK) are stimulated by ABA and different stress signals (Wrzaczek and Hirt, 2001; Ludwig et al., 2004). Gain‐ and loss‐of‐function findings have shown that signaling pathways resulting in cold, drought, salt, and tolerance are controlled through specific CDPK isoforms (Ludwig et al., 2004). Overexpression of Ca^2+‐^dependent protein kinase enhances the cold and salt/drought tolerance in rice plants (Saijo et al., 2000). *HORVU2Hr1G118320* gene encodes phosphatidylinositol kinase protein was previously found to affect cold response in the barley VIR collection (Sallam et al., 2021), maize (*Zea mays*) and *Arabidopsis thaliana* (Wang et al., 2018).

On chromosome 3H, we found an SNP located within the *HORVU3Hr1G064120* gene that encodes the Alpha/beta hydrolase domain-containing protein 13 associated with F_V_, F_V_/F_0_. The α/β-hydrolase domain (ABHD) proteins are conserved in all organisms and belong to the α/β-hydrolase (ABH) superfamily. ABH family is involved in various processes including cell signaling, energy metabolism, growth, and development (Mindrebo et al., 2016), and in response to salinity stress (Liu et al., 2014). A few of α/β-hydrolase fold enzymes, for example, esterase, phospholipase D, and prolyl oligopeptidase (POP5) play a key role in responses to several abiotic stress including drought, salt, and chilling in addition to ABA signaling of plants (Wang, 2002; Hong et al., 2010; Tan et al., 2013; Liu et al., 2014).

On chromosome 3H, the detected SNP linked with F_V_F_0_ was found within the *HORVU3Hr1G059320* gene, which encodes V-type proton ATPase subunit E. V-ATPases are complexes of membrane-embedded proteins that function as ATP hydrolysis-driven proton pumps. V-ATPase maintains the pH and the regulates acidifying of intracellular compartments. In some cell types, it is aimed at the plasma membrane, where it regulates acidifying the extracellular environment (Vasanthakumar and Rubinstein, 2020). It was found that the survival of the cells was based strongly on adjusting or maintaining the V‐ATPase activity under cold stress conditions (Dietz et al., 2001). Yoshida et al., (Yoshida et al., 1999), reported that plant sensitivity to low temperatures leads to an increase in frost and chilling hardiness including three vacuolar events related to V‐ATPase activity. (a) Chilling stress leads to the suppression of V‐ATPase activity. (b) Correspondingly, the formation of pH gradients is obstructed. (c) Then membranes adjusted their fluidity by improving the membrane content of unsaturated fatty acids. Evidence revealed that the association between stress injury and cytoplasm acidification was inveterate in studies relating V‐ATPases from chilling‐tolerant species for instance pea and chilling‐sensitive species like mung bean (Hotsubo et al., 1998). The expression level of the wheat *E* subunit of the *V-type H^+^-ATPase* gene was increased by cold, drought, salt, and exogenous ABA treatment (Zhang et al., 2014). An *in vitro* study found that chilling rice plants at 10°C increased the vacuolar-type ATPase activity (Orr et al., 1995). Cold-tolerant *Arabidopsis thaliana* and *Brassica napus* exhibited an upregulation in both proteins and subunit A mRNA in response to chilling at 2°C (Apse et al., 1999).

Another significant SNP was detected on chromosome 3H within the *HORVU3Hr1G061690* gene encoding the protein DEHYDRATION-INDUCED 19 associated F_V_F_0_. The Arabidopsis AtDi19 is a dehydration- induce protein that encodes a Cys2/His2 zinc-finger protein involved in high-salinity stress, ABA-independent dehydration, and light signaling events (Milla et al., 2006). *In vitro* assay showed that AtDi19-related proteins were phosphorylated through calcium-dependent protein kinases (CDPKs). These findings reveal that the post-translational modification could be significant in controlling the function of the AtDi19 (Milla et al., 2006). The Arabidopsis AtDi19-3 is also a transcriptional activator that participates in plant response to drought, salinity, ABA, and H_2_O_2_ events (Qin et al., 2014). In our study, we showed that the barley DEHYDRATION-INDUCED 19 is associated with chlorophyll fluorescence-related traits under low-temperature stress.

Another important genomic region F_V_F_0_ is on chromosome 3H, where their SNP is located within the *HORVU3Hr1G062030* gene that encodes ROP guanine nucleotide exchange factor 5. Guanine nucleotide exchange factors (RopGEFs) are activators of small GTPase proteins named ROPs in plants in turn regulate different cellular processes ranging from control growth to plant responses to environmental stimuli (Berken et al., 2005). The *Arabidopsis* RopGEF1 function as a negative regulator of signal transduction through the plant hormone ABA (Li et al., 2018). RopGEF1 was phosphorylated by calcium-dependent protein kinases CPK4. CPK4 stimulates RopGEF1 degradation. CPK4 also inhibits RopGEF1 activities in hairy root growth (Li et al., 2018). The *RopGEF1* (*HORVU3Hr1G085680.4*) gene expression is upregulated in salt-tolerant genotypes, which might be associated with salt stress (Chen et al., 2022). Taken together, our results could provide the basis for an advanced study into the function of barley RopGEF1 in cold acclimation tolerance.

Finally, an additional SNP was founded on chromosome 3H, within the *HORVU3Hr1G063220* gene that encodes subunit 3 of the splicing factor 3a protein complex (Splicing Factor 3A subunit 3) associated with F_V_F_0_. The *Arabidopsis* ROA1/RBM25 gene has been identified as a splicing factor required for the splicing of transcripts from several ABA signal transduction pathway genes (Zhan et al., 2015). The Arabidopsis *STA1* gene encodes a cold-induced pre-mRNA splicing factor, and *sta1-1* is cold-sensitive and defective in the splicing of the cold-induced *COR15A* gene. The splicing factor mutant *sta1-1* mutant displays that the STA1 protein regulates the splicing pattern and stability of some genes related to abiotic stress (Lee et al., 2006). The *sta1-1* and *rdm16-1* mutants exhibited hypersensitivity to ABA and salt stress in seed germination (Lee et al., 2006). In response to environmental stresses, mainly cold stress, plants modified their genome-wide alternative splicing profiles (Iida et al., 2004). Some plant hormones and environmental stresses, including cold and salt, dramatically adjust their expression and splicing profile of many important pre-mRNA splicing regulators mechanisms (Palusa et al., 2007).

The identified GO terms were related to molecular mechanisms of causal photosynthetic responses and low-temperature tolerance in barley. GO findings showed that hydrolase activity, carbohydrate metabolic process, nuclear-transcribed mRNA catabolic process, no-go mRNA decay, and protein kinase activity were enriched by identified genes linked with photosynthetic traits under cold acclimation conditions. Also, our results indicated that the zinc ion binding, protein phosphorylation, ATP binding, and guanyl-nucleotide exchange factor activity GO terms (**Table 4**) were enriched under cold acclimation conditions. These results indicate that barley plants have initiated growth and repaired damaged tissues under cold acclimation conditions. Recently a study of meta-analysis study determined the GO that plays a key role in the mechanism of barley responses to cold stress such as guanyl-nucleotide exchange factor activity, the mRNA surveillance pathway and starch and sucrose metabolism (Alamholo and Tarinejad, 2022).

Taken altogether, our functional annotation showed identified loci that were either within the known or close genes that play key roles in the photosynthetic signaling pathways, ABA signaling events, antioxidant biosynthesis, and posttranslational signals transduction. Overall, these outcomes suggest that various biological processes are involved in cold stress responses as well as post-transcriptional modification and epigenetics-mediated changes that may play essential roles in spring seedling responses to cold stress.

## 5 Conclusion

In this study, 96 spring barley genotypes were evaluated for chlorophyll fluorescence-related traits before and after the cold acclimation conditions. The genotypes were genotyped using the Barley 9K iSelect SNP Array. Our principal conclusions are the following (1) Significant phenotypic variation among genotypes under low-temperature stress. (2) Several genomic regions are associated with chlorophyll fluorescence under cold stress. (3) GWAS analysis indicated that a total of two and fifty markers were significantly associated with chlorophyll fluorescence-related traits before and after the cold stress treatment, respectively. Thirty-nine significant QTNs and thirteen annotated candidate genes were identified. We first identified the genes having suggestive and significant GWAS SNPs and discovered several potential candidate genes underlying cold acclimation stress and/or chlorophyll fluorescence trait variation. Furthermore, the candidate genes were discovered around the significant SNPs mainly on chromosomes 1H, 3H, and 6H for cold-related-traits. Most of the candidate genes participate in plant response to abiotic stress at the post-transcriptional modification level including, for example, abscisic acid (ABA) signaling, hydrolase activity, protein kinase, and transduction of environmental signal transduction at the posttranslational modification levels. (4) Novel biparental populations (RIL_2_-_5_) developed from a cross between spring x winter type which can be used to identify the new QTL for low-temperature tolerance in the spring barley in the future. Overall, our results provide fresh insight into potential low-temperature tolerance mechanisms in barley and the possibility of marker-assisted selection in the future.

## Data availability statement

The datasets presented in this study can be found in online repositories. The names of the repository/repositories and accession number(s) can be found below: https://figshare.com/, https://figshare.com/ndownloader/files/38616278.

## Author contributions

AE developed the article concept. LC-C prepared the material for genotyping. LC-C and JS physiological data collection and interpretation. AB provided the 91 varieties/breeding lines. AB made available the SNPs data. AE performed bioinformatics, data analyses, and interpretation of the results. AE wrote the manuscript. TK and AH reviewed the manuscript. All authors have read and agreed to the published version of the manuscript.
