# Supplementary material for: Genome-wide association analysis provides insights into the genetic basis of photosynthetic responses to low-temperature stress in spring barley
Source: Front Plant Sci. 2023 Jun 6;14:1159016. doi: 10.3389/fpls.2023.1159016 (PMC10279893; doi:10.3389/fpls.2023.1159016)
Supplement: Supplementary file 1 [file DataSheet_1.pdf]

Supplementary Material

Genome-wide association analysis provides insights into the genetic basis of photosynthetic responses to low-temperature stress in spring barley

Ammar Elakhdar[1,2,*], Jan J. Slaski[3], Takahiko Kubo[2], Aladdin Hamwieh, Aaron D. Beattie[4], Ludovic J.A. Capo-chichi[5]

*** Correspondence:**
Ammar Elakhdar
a.elakhdar@kyudai.jp

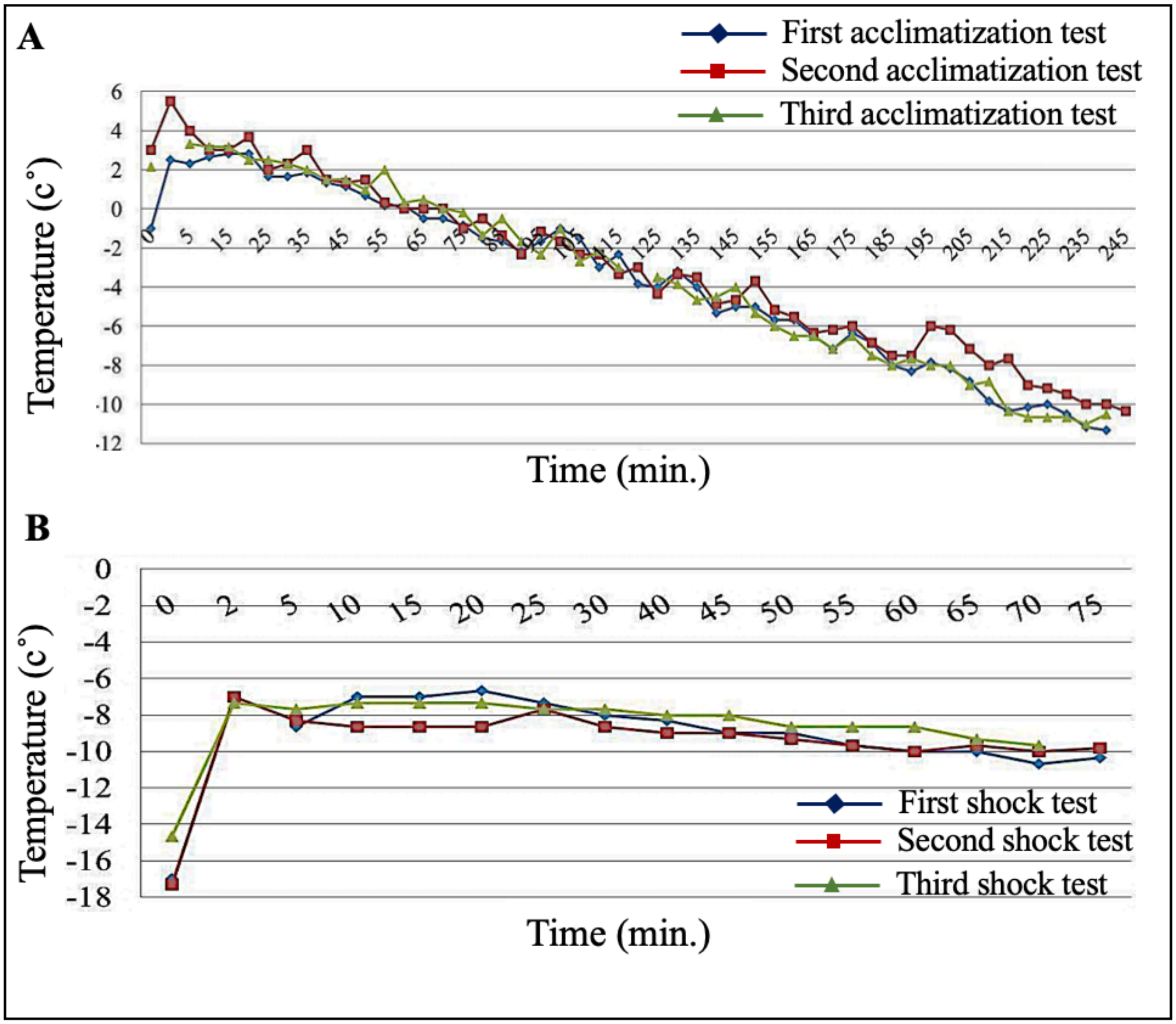

Supplementary Figure 1: Temperature variation during cold acclimation treatment (A) and freezing shock (B) of 96 spring barley lines and 4 winter barley lines at the three-leaf stage.

HORVU1Hr1G087520

SUMO transferase activity [GO:0019789] | zinc ion binding [GO:0008270]

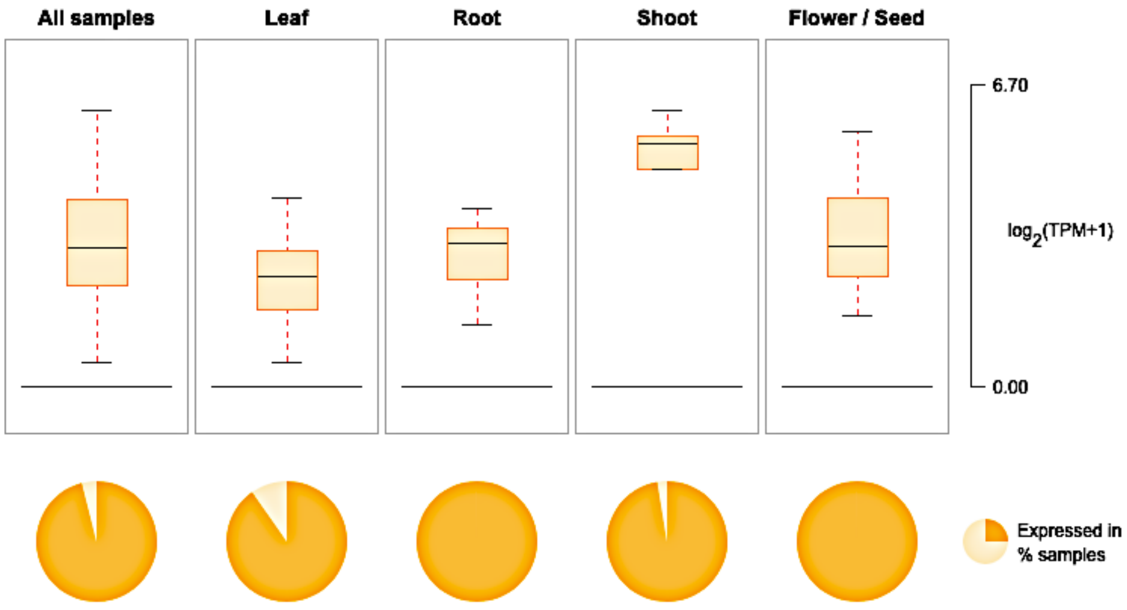

HORVU3Hr1G071210

hydrolase activity, hydrolyzing O-glycosyl compounds [GO:0004553]

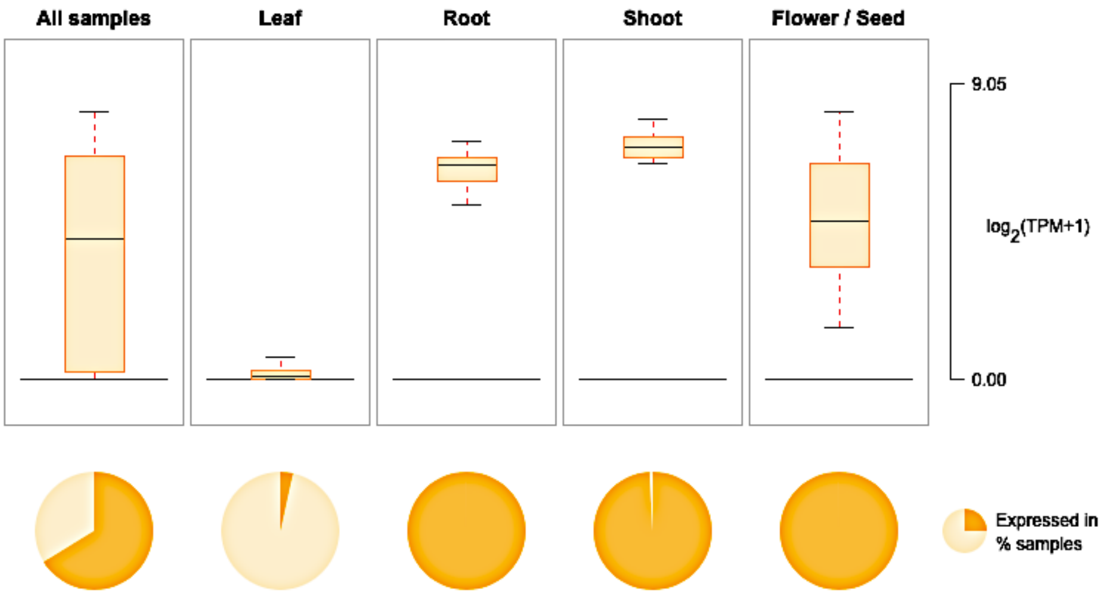

HORVU3Hr1G071240

ATP binding [GO:0005524] | protein kinase activity [GO:0004672]

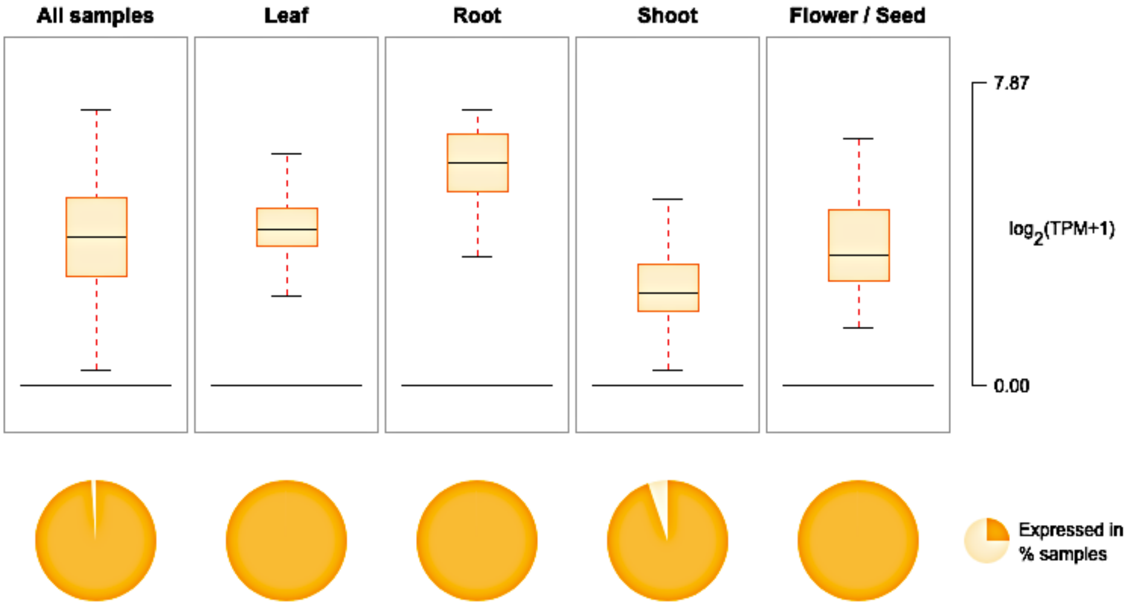

HORVU6Hr1G005960
DNA binding [GO:0003677] | protein heterodimerization activity [GO:0046982]

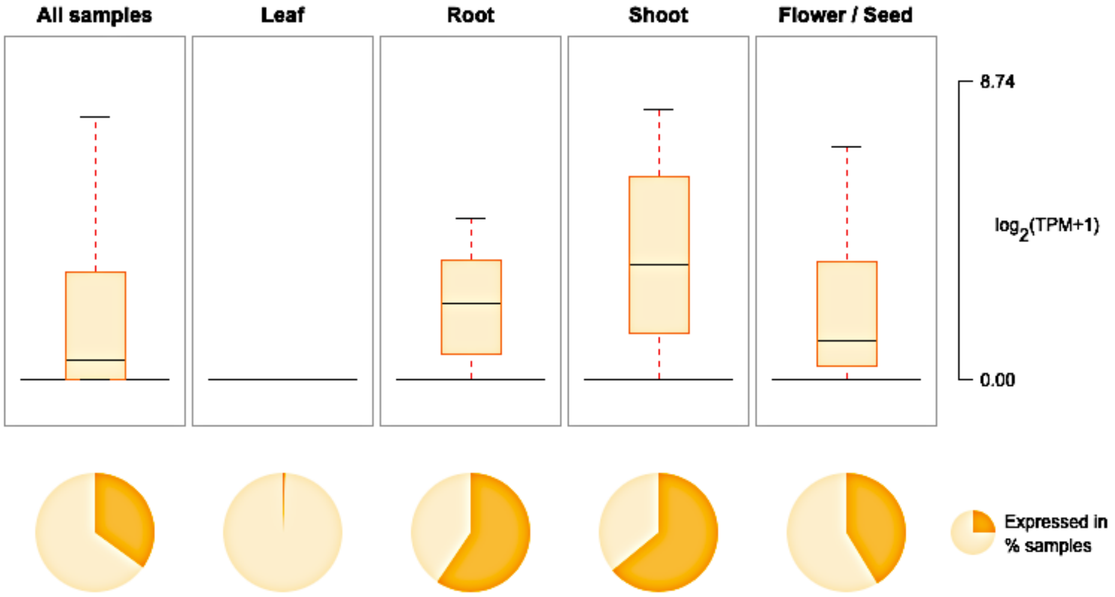

HORVU3Hr1G059320
ATP hydrolysis coupled proton transport [GO:0015991]

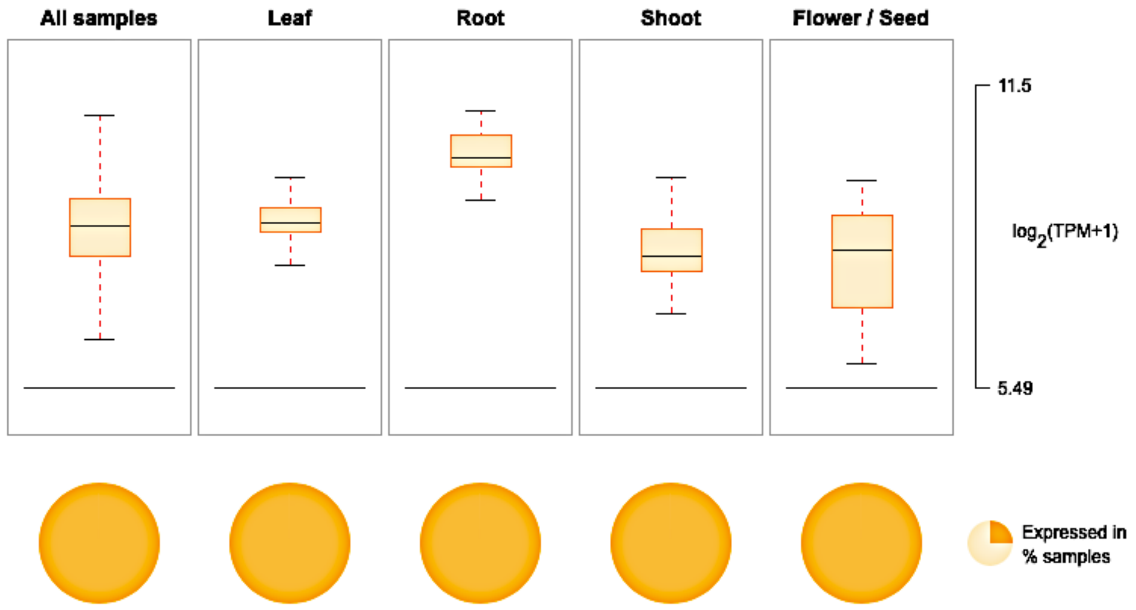

HORVU3Hr1G061690
Protein DEHYDRATION-INDUCED 19 homolog 3

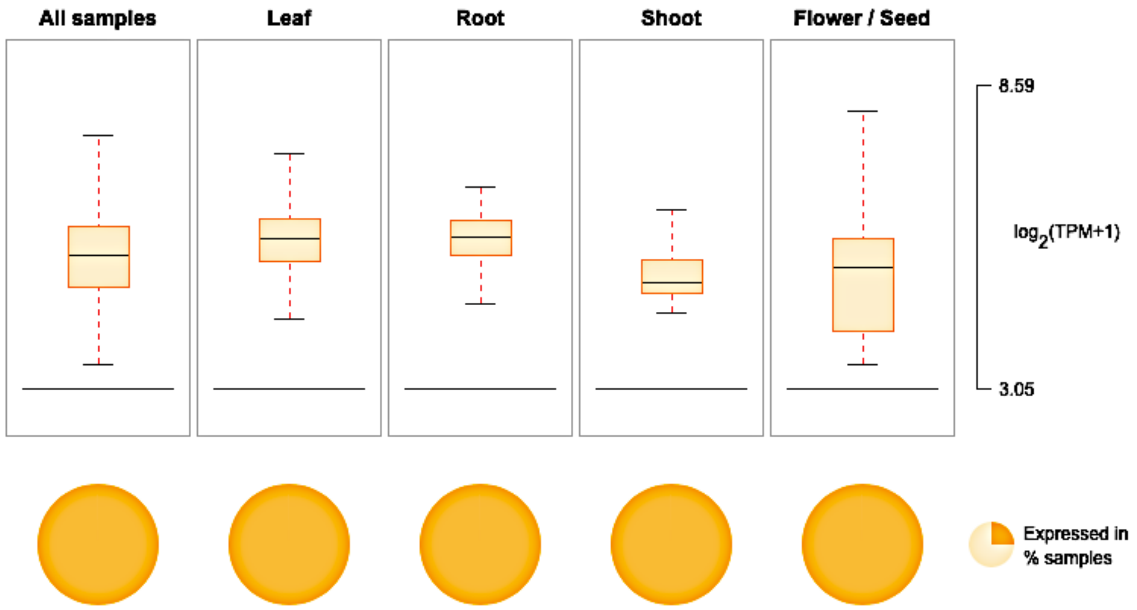

HORVU3Hr1G062030
Rho guanyl-nucleotide exchange factor activity [GO:0005089]

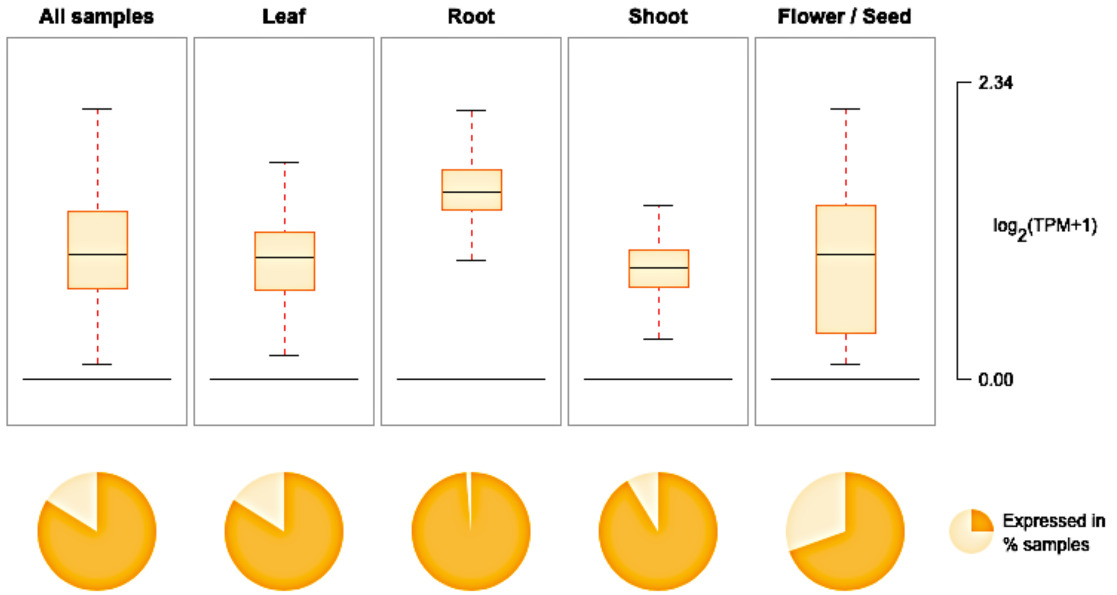

HORVU3Hr1G063220
mRNA splicing, via spliceosome [GO:0000398] | regulation of embryo sac egg cell differentiation [GO:0045694]

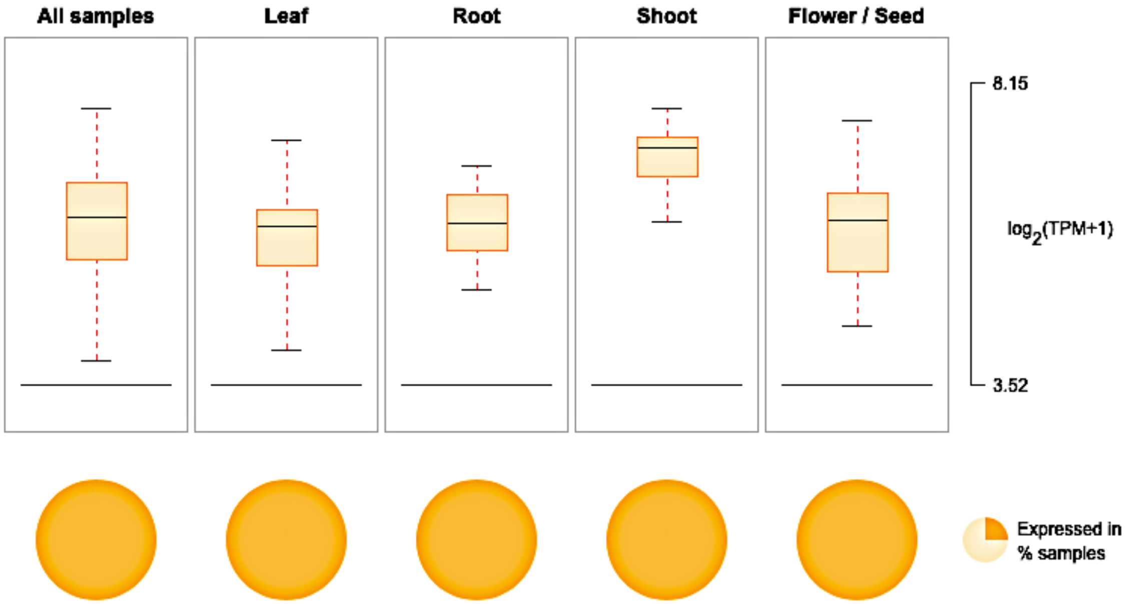

HORVU1Hr1G085880
signal transduction [GO:0007165]

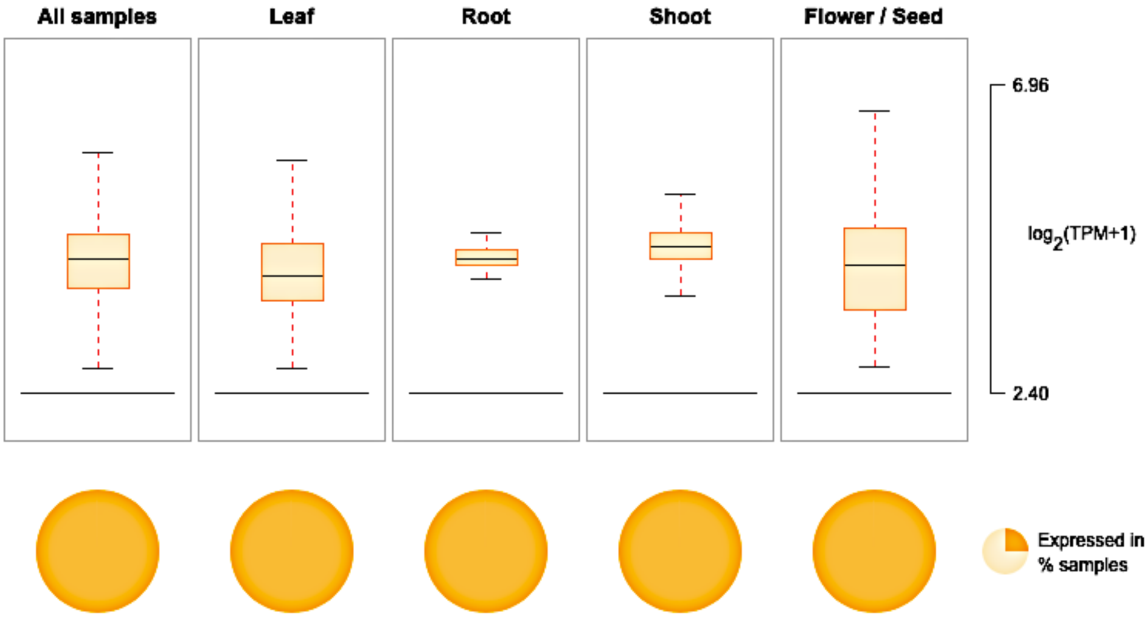

Supplementary Figure 2: Gene co-expression for the identified genes was revealed form the Global gene co-expression networks (GCNs) database PlantNexus (https://plantnexus.ohio.edu).

Supplementary Table 1. List of crosses made between spring and winter barley types.

Cross		Spring genotypes		Winter genotypes
3	♀	Meredith	♂	02Ab431
4	♀	AC Metcalfe	♂	02Ab08-X05W061-208
5	♀	TR643	♂	02Ab671
6	♀	TR990	♂	02Ab431
11	♀	TR162	♂	02Ab671
12	♀	TR490	♂	02Ab671
13	♀	TR05912	♂	02Ab08-X05W061-208
14	♀	CDC Reserve	♂	02Ab431
15	♀	Meredith	♂	02Ab08-X05W061-208
16	♀	TR643	♂	02Ab08-X05W061-208
17	♀	TR490	♂	02Ab08-X05W061-208
18	♀	AC Metcalfe	♂	02Ab431
		**Winter genotypes**		**Spring genotypes**
1	♀	02Ab669	♂	TR990
2	♀	02Ab431	♂	TR5287
7	♀	02Ab08-X05W061	♂	CDC Reserve
8	♀	02Ab431	♂	AC Metcalfe
9	♀	02Ab431	♂	Benthey
10	♀	02Ab08-X05W061	♂	AC Metcalfe
